# Rotation of the Fla2 flagella of *Cereibacter sphaeroides* requires the periplasmic proteins MotK and MotE that interact with the flagellar stator protein MotB2

**DOI:** 10.1371/journal.pone.0298028

**Published:** 2024-03-20

**Authors:** Fernanda Vélez-González, Arely Marcos-Vilchis, Benjamín Vega-Baray, Georges Dreyfus, Sebastian Poggio, Laura Camarena

**Affiliations:** 1 Instituto de Investigaciones Biomédicas, Universidad Nacional Autónoma de México, Mexico City, Mexico; 2 Instituto de Fisiología Celular, Universidad Nacional Autónoma de México, Mexico City, Mexico; East Carolina University Brody School of Medicine, UNITED STATES

## Abstract

The bacterial flagellum is a complex structure formed by more than 25 different proteins, this appendage comprises three conserved structures: the basal body, the hook and filament. The basal body, embedded in the cell envelope, is the most complex structure and houses the export apparatus and the motor. *In situ* images of the flagellar motor in different species have revealed a huge diversity of structures that surround the well-conserved periplasmic components of the basal body. The identity of the proteins that form these novel structures in many cases has been elucidated genetically and biochemically, but in others they remain to be identified or characterized. In this work, we report that in the alpha proteobacteria *Cereibacter sphaeroides* the novel protein MotK along with MotE are essential for flagellar rotation. We show evidence that these periplasmic proteins interact with each other and with MotB2. Moreover, these proteins localize to the flagellated pole and MotK localization is dependent on MotB2 and MotA2. These results together suggest that the role of MotK and MotE is to activate or recruit the flagellar stators to the flagellar structure.

## Introduction

The bacterial flagellum is a complex structure that extends from the cytoplasm, trough the cell envelope, and into the extracellular space. It is divided in the basal body, the hook, and the filament. In several enterobacteria such as *Escherichia coli* the filament is formed by multiple subunits of the FliC protein and reaches a length of 15 to 20 μm. The hook connects the filament with the rod that expands throughout the periplasmic space. The basal body is the most complex structure and comprises the flagellar export apparatus as well as the rotative part of the motor [[reviewed in [1–6]]. The export apparatus is composed of 5 membrane proteins, FliPQR, FlhA and FlhB that are located at the central part of the MS ring (supra-membrane ring) that is formed by subunits of the FliF protein [7–11]. On the periplasmic side of the MS ring, the rod is assembled by the sequential addition of the FliE, FlgB -C, -F and -G proteins [12–17]. The assembly of this structure requires the action of a scaffolding protein and a peptidoglycan-hydrolase or lytic transglycosylase that facilitates the penetration of the cell wall [18]. In *E*. *coli*, these activities are carried out by discrete domains of the protein FlgJ [19, 20]. When the rod reaches the outer membrane the protein FlgJ is released from the tip of the rod into the milieu and the assemble of the hook initiates [21]. The rod is surrounded by two rings that presumably act as a bushing during flagellar rotation, the P-ring is assembled near the cell wall, and the L-ring in the outer membrane [14, 16, 22–24]. On the cytoplasmic side of the MS ring, the C-ring is assembled; this ring consists of three cytoplasmic proteins, FliG, FliM and FliN [25–31], and is essential for torque generation along with the stator proteins MotA and MotB. The interaction of the stator with FliG allows the transformation of the ion-motive force across the membrane into rotational force [32–36]. The C-ring is also involved in the rotational switching controlled by the chemotactic system [37–39].

MotA and MotB form a complex constituted by two subunits of MotB surrounded by 5 subunits of MotA [40, 41]. This complex conduct protons across the membrane using a conserved Asp residue localized in the transmembrane region of MotB [42–44]. Depending on the species, from 11 to 18 MotA/MotB complexes are arranged around the rotor [45–48]. Before their association with the rotor, the proton channel is inactive given the interaction of a short amphipathic helix of MotB and the outer leaflet of the inner membrane [49]. Upon association with the flagellar rotor, the periplasmic domain of MotB is rearranged adopting an extended conformation in which the peptidoglycan domain of MotB reaches the cell wall and the proton channel is unplugged [50–52]. For several species, it has been reported that the membrane protein FliL contributes to this process and motility is strengthened [53–57]. Similarly, the *Vibrio*-specific proteins MotX and MotY form an additional ring in the periplasmic space and MotX directly interacts with the MotB homologue, PomB, favoring the recruitment of the stator complexes [58–62]. In this regard, observation of the flagellar structure *in situ* has revealed that the flagellar motors of many bacteria have additional components that are absent in the prototypical motor of *E*. *coli* and *Salmonella enterica*. These components embellish different parts of the prototypical motor. However, their function and the identity of the proteins that form these additional structures remains to be fully discovered [1, 46, 48, 63–65].

The alpha-proteobacterium *Cereibacter sphaeroides* possesses two different flagellar systems [66]. The *fla1* genes are responsible for the assembly of a single subpolar flagellum [67], whereas the *fla2* genes enable the assembly of several polar flagella [68], with the number varying from 2 to 9 flagella per cell and an average of 4.5 **±** 1.79 [69]. Under the growth conditions commonly used in the laboratory, the *fla1* genes are constitutively expressed and the *fla2* genes are transcriptionally inactive [68]. Phylogenetic analyses have shown that the *fla1* genes originated from a horizontal transfer event whereas the *fla2* genes are the vertically inherited genes in the α-proteobacteria linage [68]. So far, the Fla2 flagellum has been studied in mutant strains carrying two different mutations, one prevents the expression of Fla1 by removing the gene encoding for the Fla1-master regulator FleQ, and the other, is a gain of function mutation in CckA or a deleterious mutation in its negative regulator Osp [70–72].

Fla1 and Fla2 rotate unidirectionally, and reorientation is achieved by controlling the frequency of short stop events [67, 73]. During smooth swimming periods, cells reach average velocities of 27 to 50 μm/s when swimming with the Fla1 flagellum [74–77], and approximately 61 μm/s with Fla2. Complex chemotactic systems control each of these flagellar motors and noteworthily, the expression of the chemotactic genes is coordinated by the transcriptional factors that control the expression of their cognate flagellar system [78]. In contrast to the Fla1 system, in which the activator protein FleQ only activates the expression of the *fla1*genes and the chemotactic genes that control its rotation [78], the transcriptional factor CtrA activates the expression of the *fla2* genes, the cognate chemotactic genes, as well as the expression of approximately 200 additional genes [72]. Therefore, CtrA orchestrates a global response that encompasses the assembly of the Fla2 flagella, gas vesicle formation, activation of genes involved in stress responses, and repression of photosynthetic genes. In this scenario, Fla2 motility is coordinated with other cellular responses that likely favor a particular lifestyle. It has been speculated that the coordinated expression of gas vesicles together with a competent Fla2 motility system could be related with an effective colonization of the surface layer of a water body [72].

Electron microscopy as well as genetical and biochemical studies have shown that the Fla1 motor includes additional components that are absent in the canonical motor of *E*. *coli* and *S*. *enterica* but that are essential for flagellar formation or motor rotation, such as the H-ring formed by the FlgT protein which surrounds the L and P-rings [79], the *Cereibacter*-specific MotF protein [80] and the lipoprotein FlgP [81]. In contrast to Fla1, electron microscopy images of the isolated Fla2 flagella showed a structure highly similar to that of *S*. *enterica* and *E*. *coli*; additionally, mass spectrometry analysis of these samples, revealed no additional proteins beyond the known flagellar proteins present in the mentioned enteric bacteria [66, 69]. However, the *fla2* locus does contain several open reading frames (orfs) encoding hypothetical or conserved proteins that have not been studied. Therefore, it is relevant to determine if these putative proteins are involved in the function or assembly of the flagellum.

RSWS8N_12055 and RSWS8N_12065 are located within the *fla2* locus, and their possible role in the functioning of the Fla2 flagella has not been determined [82]. Currently, there are no reports for the role of the product of RSWS8N_12065; however, the product of RSWS8N_12055 is homologous to MotE from *Sinorhizobium meliloti* [83]. In this bacterium, it was reported that MotE is a periplasmic protein that is required for flagellar rotation. The main role of MotE is to interact and protect the periplasmic protein MotC from degradation [83]. In *S*. *meliloti* the MotC protein is encoded downstream of *motB* and the paralyzed phenotype of the *motB* and *motC* mutants is only recovered when *motB* and *motC* are expressed together, suggesting that an appropriated stoichiometry of these proteins is needed for motility [84]. From these results it was proposed that MotE acts as the chaperone of MotC, and that MotC could interact with MotB to control flagellar rotation [83, 84]. Interestingly, *C*. *sphaeroides* does not have a MotC homologue, therefore the function of MotE in *C*. *sphaeroides* is not clear.

In this work we show that MotE of *C*. *sphaeroides* is essential for flagellar rotation and interacts with another periplasmic protein that is also essential for flagellar rotation encoded by RSWS8N_12065, from here on named as MotK. Protein-protein interaction analysis revealed that these proteins interact with MotB2 and localization of MotK-sfGFP requires of the stator proteins MotA2 and MotB2. Therefore, we propose that the paralyzed phenotype associated with the absence of MotE and MotK could be explained by a defect in the recruitment or functioning of the flagellar stators.

## Materials and methods

### Bacterial strains, plasmids, and growth conditions

Bacterial strains and plasmids used in this work are listed in Table 1. *C*. *sphaeroides* was grown at 30°C in Sistrom’s minimal medium [85] with 15 mM succinic acid for maintenance or strain selection and with 0.2% casamino acids as carbon source for all the experimental procedures. Photoheterotrophic liquid cultures were grown under continuous illumination in completely filled screw-cap tubes. Heterotrophic liquid cultures were incubated in the dark with orbital shaking at 200 rpm. Plates were prepared using Sistrom’s minimal medium with 1.5% agar. *E*. *coli* was grown at 37°C in LB medium [86]. When needed, antibiotics were added at the following concentrations: for *C*. *sphaeroides*, spectinomycin 100 μg/ml; kanamycin 25 μg/ml; hygromycin 20 μg/ml for liquid cultures and 150 μg/ml for plates; tetracycline 1 μg/ml. For *E*. *coli*, ampicillin 100 μg/ml; kanamycin 50 μg/ml; tetracycline 12 μg/ml; spectinomycin 100 μg/ml; hygromycin 20 μg/ml for liquid cultures and 150 μg/ml for plates; nalidixic acid 20 μg/ml.

**Table 1:** Strains and plasmids used in this work.

Strain	Relevant characteristics	Reference
***Cereibacter sphaeroides***		
WS8N	wild-type strain Nal^R^	[87]
AM1	WS8N derivative Δ*fleQ*::kan cckA_L391F_	[88]
EA1	AM1 derivative; Δ*ctrA*::*aadA*	[70]
IM1	AM1 derivative; *motK*::*aadA*	This work
IM3	AM1 derivative; Δ*motK*::hyg *motB2*::*aadA*	This work
IM4	AM1 derivative; Δ*motK*::hyg *motA2*::*aadA*	This work
IM6	AM1 derivative; *motB2*::*aadA*	This work
IM7	AM1 derivative; *motA2*::*aadA*	This work
FV1	AM1 derivative; *motE*::*aadA*	This work
FV2	AM1 derivative; ΔmotK::hyg	This work
***Escherichia coli***		
LMG194	Protein expression strain	Invitrogen
TOP10	Cloning strain	Invitrogen
Rosetta	Protein expression strain	Novagen
**Plasmids**		
pBAD/His A or B	Expression vectors of His6x-tagged proteins, Ap^R^	Invitrogen
pBAD_motB2	pBAD/HisB expressing His6x-MotB2	This work
pBAD_motK	pBAD/HisA expressing His6x-MotK	This work
pET28a	Expression vector for His6x-tagged proteins, Kan^R^	Novagen
pET28_fliL2	pET28a expressing His6x_FliL2	This work
pET28_motE	pET28a expressing His6x_MotE	This work
pGEX-4T-2	Expression vector for GST gene fusion, Ap^R^	Cytiva
pGEX_motE	pGEX-4T-2 expressing GST-MotE	This work
pIJ963	Plasmid source of the Hyg cassette	[89]
pJQ200mp18	Suicide vector for *C*. *sphaeroides*, Gm^R^	[90]
pJQ_motA2::aadA	pJQ200 carrying *motA2*::*aadA*	This work
pJQ_motB2::aadA	pJQ200 carrying *motB2*::*aadA*	This work
pJQ_motK::aadA	pJQ200 carrying *motK*::*aadA*	This work
pJQ_motE::aadA	pJQ200 carrying *motE*::*aadA*	This work
pJQ_motK::hyg	pJQ200 carrying Δ*motK*::hyg	This work
pPIRL	Vector that encodes tRNAs for rare codons; Cm^R^	[91]
pRK415	Expression vector used in *C*. *sphaeroides*, Tc^R^	[92]
pRK_motE	pRK415 expressing MotE	This work
pRK_motEC20A	pRK415 expressing MotE_C20A_	This work
pRK_motK	pRK415 expressing MotK	This work
pRK_MotE-sfGFP	pRK415 expressing MotE-sfGFP	This work
pRK_MotK-sfGFP	pRK415 expressing MotK-sfGFP	This work
pTB263	Plasmid encoding sfGFP	[93]
pTZ18R/19R	Cloning vectors, Ap^R^	Amersham
pTZ_motA2	PCR of *motA2* cloned in pTZ18R	This work
pTZ_motB2	PCR of *motB2* cloned in pTZ18R	This work
pTZ_motK	PCR of *motK* cloned in pTZ18R	This work
pTZ_motE	PCR of *motE* cloned in pTZ18R	This work
pTZ_motA2::aadA	pTZ18R carrying *motA2*::*aadA*	This work
pTZ_motB2::aadA	pTZ18R carrying *motB2*::*aadA*	This work
pTZ_motK::aadA	pTZ18R carrying *motK*::*aadA*	This work
pTZ_motE::aadA	pTZ18R carrying *motE*::*aadA*	This work
pTZ_motK::hyg	pTZ18R carrying Δ*motK*::hyg	This work
pTZ_motE_C20A_	pTZ18R carrying *motE*_C20A_	This work

### Swimming evaluation

Swimming proficiency was determined by microscopic observation of cells grown in liquid medium, or by using soft-agar plates containing Sistrom’s minimal medium with 0.2% casamino acids and 0.2% agar. These plates were inoculated with a 2 μl sample of a fresh saturated culture spotted on the surface, and incubated anaerobically in a transparent polycarbonate anaerobic jar using the BD GasPak EZ anaerobe container system sachets and illuminated with incandescent bulbs (two at 75 W) for 72 h. For the experiments in Fig 2, swimming was evaluated at least in three independent experiments. Representative images are shown.

### Isolation of mutant strains

Mutagenesis of *motK*, *motB2*, and *motA2* genes was done by allelic replacement using the suicide plasmid pJQ200 mp18 [90], carrying the suitable mutant allele. For this, the target gene including 100 bp upstream and downstream of the coding region was amplified by PCR using the appropriate pair of oligonucleotides (S1 Table). The PCR products of 2,163, 1,038, and 1,070 bp obtained for *motK*, *motB2*, and *motA2* were cloned into pTZ18R plasmid. The resultant plasmids were digested using a unique restriction site present in the coding region of these genes (HincII for *motK*, BglII for *motB2*, and XhoII for *motA2*) and ligated with a non-polar spectinomycin cassette (*aadA*) obtained from pMW5 plasmid [94]. A mutant allele of *motK* interrupted with an hygromycin resistance cassette (hyg) was obtained by digesting pTZ_motK with ClaI and EcoRV; then, termini were repaired to blunt ends with T4 DNA polymerase and ligated with a hygromycin (hyg) resistance cassette previously obtained by PCR from pIJ963 plasmid. The mutant alleles were subcloned in pJQ200mp18. Mutagenesis of *motE* was carried out by cloning the PCR product of 1160 bp obtained with the oligonucleotides FW_1314 and Rv_1315 Xba in pTZ18R; this plasmid was digested with EcoO1091, termini repaired to blunt ends with T4 DNA polymerase and ligated with a non-polar spectinomycin cassette. The DNA fragment carrying the mutant allele *motE*::*aadA* was subcloned in the suicide plasmid pJQ200mp18.

To obtain the mutant strains, the suicide plasmid carrying the interrupted allele was introduced to *C*. *sphaeroides* by conjugation and the double recombination events were selected as described previously [95, 96]. The presence of the correct gene replacement was verified by PCR.

### Plasmid constructs used in this work

Plasmids pRK_motK and pRK_motE were obtained by cloning in pRK415 [92] the PCR product of 2,163 bp obtained with the oligonucleotides FW-1318HindIII and REV-1318XbaI for *motK*, and the PCR product of 780 bp obtained with the oligonucleotides FW_HindIII_1315 and Rv_1315 Xba for *motE*. pRK_motE-sfGFP was generated by cloning together the PCR products of *motE* (660 bp) and superfolder *gfp* (sf*gfp*) (741 bp) obtained using the oligonucleotides FW_HindIII1315 and Rv sf 1315 for *motE*, and Fw SF GFP and Rv SF GFP for sf*gfp*, the resultant product was cloned into pRK415 plasmid. pRK_motK-sfGFP was generated by cloning together the PCR products of *motK* and sf*gfp* obtained with the oligonucleotides FW-1318HindIII y REV1318Sma-S/Stop for *motK* and FW-sfGFP-Sma S/ATG and REV-sfGFP EcoRI for sf*gfp*, the resultant product was cloned in pRK415 plasmid.

### Site directed mutagenesis

Site-directed mutagenesis of *motE* was carried out by overlap extension PCR [97] using the oligonucleotides FW_1314 and 1315 CysAla for the upstream region, and 1315 CysAla1 and 15DH-RvEco 1315 for the downstream region. pTZ_motE was used as template for the first amplification reaction. The resultant product of the second amplification reaction was cloned into pTZ18R. The presence of the desired mutation was verified by sequencing. The DNA fragment carrying the mutant allele was subcloned into pRK415 plasmid.

### Analysis of MotK and MotE

The primary sequence of the MotK and MotE proteins was analyzed using TOPCONS to identify a potential signal peptide [98]. Structure predictions of these proteins were obtained from AlphaFold [99, 100]. The N-terminal region of MotK and other homologues was further analyzed using I-Tasser and Swiss-Model [101–103]. Synteny was evaluated using the Genomic Context Visualizer (GeCoViz https://gecoviz.cgmlab.org) [104].

### Electron microscopy

Bacterial cells were examined by transmission electron microscopy using a 1 μl sample of an exponentially grown culture applied on a Formvar coated grid. Samples were negatively stained with 1% uranyl acetate and observed with a JEM-1200EXII microscope (Jeol, Tokyo, Japan).

### Fluorescence microscopy

2 μl of an exponentially growing culture was placed on an agarose-coated slide and observed by epifluorescence microscopy. Images were taken with a Hamamatsu Orca-ER camera and a Nikon E600 microscope. Flagella was detected by immunocytochemistry using anti-FlgE2 antibody labeled with Zenon Alexa Fluor 546, as reported previously [70]. Images were processed with ImageJ [105]. Microscopic observations were done in three independent occasions for the experiments corresponding to Figs 9 to 12. Representative images are shown. Published protocols were followed to determine the fluorescence intensity of the foci formed by MotE-sfGFP or MotK-sfGFP with respect to the background [106, 107]; briefly, the grey values of a small circular area were obtained for the polar region (focus); to this value the grey value of a region inside the cell was subtracted. The average of the resulting values from 120 cells was obtained. Significance of the difference between the populations was assessed by one-way analysis of variance (ANOVA) to compare data across different strains.

### Proteolytic susceptibility assay

Cells from an exponentially growing culture (5 ml) were collected at 16,100 xg for 5 min, washed twice with Tris-HCl buffer (Tris 10 mM pH 7), and resuspended in 500 μl of Tris-HCl buffer containing 20% sucrose. A small aliquot to be used as a negative control was taken before the addition of lysozyme (0.5 mg/ml) and EDTA (50 μM). After 15 min of incubation at 37°C, the sample containing the spheroplast was divided in two and supplemented either with 0.1 mg/ml of Proteinase K or buffer; incubation was allowed to proceed for 15 min. To stop the reaction, phenylmethylsulfonyl fluoride (2 μM) was added and the samples were further incubated for 5 min before being boiled in 1X Laemmli sample buffer [108]. Proteins were subjected to SDS-PAGE and visualized by Western blot using the indicated primary antibodies. The experiments presented in Fig 3 were performed at least three times, a representative image is shown.

### Protein overexpression and purification

To overexpress the mature form of MotK the coding region of *motK* was amplified using total DNA from WS8N and the oligonucleotides FW-1318Sac-pQE30 y REV1318Hind-pQE30. The 1897 bp product was cloned in pBAD-HisA and transformed in LMG194/pPIRL. To purify His6x-MotK, an exponential phase culture of LMG194/pPIRL/pBAD_motK was induced with 0.002% L-arabinose, after 3 h of incubation at 37°C, cells were harvested and resuspended in 1/50 of the original volume using phosphate buffer pH 8 (50 mM Na_2_HPO_4_, 150 mM NaCl, 10 mM imidazole). Spheroplasts were formed by adding lysozyme (1mg/ml) and proteases were inhibited using cOmplete EDTA-free tablets (Roche) as recommended by the manufacturer. The sample was incubated on ice for 30 min at 4°C and lysed by sonication. Cell debris were removed by centrifugation and the supernatant was mixed with Ni-NTA agarose beads (Qiagen) and incubated for 2 h on a platform rocker. After this time, the beads were washed with phosphate buffer and 20 mM imidazole. Elution was done using phosphate buffer with 200 mM imidazole.

To overexpress the periplasmic region of MotB2, the PCR product of 685 bp obtained with the oligonucleotides FwKpnMotB2p and RvHindMotB2pstop was cloned into pBAD-HisB plasmid and transformed into LMG194/pPIRL. An exponential phase culture of LMG194/pPIRL/pBAD_motB2 was induced with 0.2% L-arabinose, after 2 h of incubation at 37°C, cells were collected and His6x-MotB2 was purified following the same protocol used to obtain MotK.

To overexpress the periplasmic domain of FliL2, the DNA region encoding this domain was amplified by PCR using chromosomal DNA from WS8N and the oligonucleotides FliLep-Sal and FliLepRv-NotI. The product of 375 bp was cloned in pTZ18R and subsequently subcloned into pET28a. An exponential phase culture of Rosetta/pET28_fliL2 was induced with 0.5 mM IPTG, after 3h of incubation at 37°C, cells were collected and FliL2 was purified following the procedure described above.

To overexpress the mature form of MotE, the coding region of *motE* was amplified using total DNA from WS8N and the oligonucleotides rsp1315fwkpn and rsp1315rvhindIII. The product of 480 bp was cloned into pQE30 but given that the protein was not expressed, the 515 bp BamHI-HindIII fragment from pQE30_motE was subcloned into pET28a and the resultant plasmid was transformed into Rosetta strain. His6x-MotE was obtained by inducing an exponential phase culture of Rosetta/pET28_motE strain with 0.5 mM IPTG during 3 h at 37°C. After incubation, cells were harvested and resuspended in Tris-HCl buffer pH 8 (50 mM Tris-HCl, 5% glycerol, 50 mM NaCl). Spheroplasts were formed by incubating the sample on ice during 30 min in the presence of 1 mg/ml of lysozyme. After lysis by sonication the soluble protein was purified following the procedure described above.

To overexpress GST-MotE fusion protein, the 523 bp BamHI-NotI fragment obtained from pET28_motE was cloned into pGEX-4T-2. An exponential culture of Rosetta strain carrying pGEX_motE was induced with 0.5 mM IPTG during 12 h at 30°C. Then, cells were harvested and resuspended in Tris-HCl buffer pH 8 (50 mM Tris-HCl, 5% glycerol, 50 mM NaCl). Spheroplasts were formed by incubating the sample on ice during 30 min in the presence of 1 mg/ml of lysozyme. After lysis by sonication and removal of the cellular debris, the soluble protein was purified using glutathione-agarose beads (Sigma-Aldrich). The sample was incubated in the presence of the beads during 1 h on ice with occasional mixing by inversion. The beads were washed with PBS buffer pH 8 and the protein was eluted using Tris-HCl buffer pH 8 containing 10 mM reduced glutathione.

### Antibodies generation and Western blot

MotK, and MotB2, antibodies were raised in rabbits according to standard procedures [109]. Two males New Zeland rabbits were inoculated two times with Freund’s complete adjuvant and 200 μg of denatured His6x-MotK protein or native His6x-MotB2. Inoculations were separated by two-week interval. Bleeding was performed after 3 weeks had passed from the second inoculation. His6x-MotE antibodies were raised in female BALB/c mice as described [109]. α-FlgE2, and α-CckA antibodies were used as previously reported [68, 70]. Western blots were done according to the method described previously [109], using the indicated primary antibody followed by incubation with HRP-or AP-conjugated secondary antibodies. Blots were developed with CDP-Star (Thermo Fisher Scientific) or SuperSignal West Pico PLUS (Pierce) and exposed to an X-ray film for visualization.

### Detection of the hook protein FlgE2 in the supernant culture

Cells from 1 ml culture were vigorously mixed for 10 min to shear off the flagella. Supernatant fractions were obtained by centrifugation (14,000Xg for 5 min at 4°C). Proteins in the supernatant were precipitated with chloroform-methanol [110] and resuspended in 20 μl of sample buffer. Cells from 1 ml culture were collected by centrifugation and the cellular fraction was resuspended in 250 μl of sample buffer. Samples (10 μl) were subject to SDS-PAGE electrophoresis, blotted and then probed with α-FlgE2 antibody. To verify the integrity of the cells, the samples were also tested with the α-CckA antibody that recognizes an intracellular protein.

### GST pulldown assay

For this assay 5 μg of GST-MotE or GST were bound to glutathione beads in PBS buffer pH 7.4 and mixed with His6x-MotK or His6x-MotB2 in a molar ratio 1:1. The mixture was incubated for 2 h mixing by gentle flickering every 10 min. The beads were collected by centrifugation at 3,000 rpm for 1 min and the supernatant was removed by aspiration. The beads were washed four times with PBS buffer. Proteins were recovered in 100 μl of 100 mM Tris-HCl pH 8 containing 10 mM reduced L-glutathione. A 15 μl sample was subjected to SDS-PAGE and analyzed by Western blot using anti-MotB2, anti-MotK or anti-GST (Sigma Aldrich) antibodies. The experiments presented in Fig 6 were performed at least three times, a representative image is shown.

### Far Western

Total cell extracts of *E*. *coli* carrying pBAD_motK, pBAD_motB2 and pET28_fliL2 were grown until exponential phase and half of the culture was induced by adding 0.002% or 0.2% arabinose for MotK or MotB2, respectively, and 0.5 mM IPTG for FliL2. After 2 h of incubation at 37 C, cells were collected and lysed in SDS-loading buffer. Samples were then subject to SDS-PAGE and transferred to a nitrocellulose membrane, according to standard procedures [109]. The blot was blocked in 10 mM Tris-HCl buffer pH 7.4 containing 150 mM NaCl, 0.05% Tween 20 and 2% non-fat milk for 2 h at room temperature; then, it was incubated with purified His6x-MotE or His6x-MotB2 (0.5 μg/ml) for 4 h at room temperature. The blot was washed and probed with anti-MotE or anti-MotB2 antibodies [111, 112]. Detection was done using a secondary antibody coupled to alkaline phosphatase (Sigma-Aldrich) and CDP-star (ThermoFisher, Scientific) according to the manufacturer’s instructions. The experiments presented in Figs 5 and 7 were performed at least three times, a representative image is shown.

## Results

### Analysis of MotE and MotK

In *C*. *sphaeroides* most of the *fla2* genes are clustered in a region of approximately 32.3 Kb present in chromosome I [82]. In this cluster we found a putative operon comprising *fliL2*, WS8N_12050, RSWS8N_12055 (*motE*), *motA2* and RSWS8N_12065 (*motK*). These genes probably form an operon given that their stop and start codons are overlapped or closely located, and the binding site of the transcriptional regulator CtrA required to express the *fla2* genes, is found upstream of *fliL2* [72, 82]. As previously mentioned, in bacteria, MotA and FliL, are involved in flagellar rotation. Homologues of the proteins encoded by WS8N_12050 (*pflI*) and RSWS8N_12055 (*motE*) have only been studied in *C*. *crescentus* and *S*. *meliloti*, respectively [83, 113]. The homologue of WS8N_12050, encoding the protein PflI, has been implicated in positioning the flagellar structure [113], and MotE, is required for flagellar rotation in *S*. *meliloti* [83]. No homologues for RSWS8N_12065 (*motK*) have so far been reported and the protein encoded by this gene does not show any conserved domains. Similar proteins are present in some species of *Paracoccaceae*; and, a BLAST search excluding *Paracoccaceae* revealed the presence of similar proteins also in species of the *Roseobacteracea* family. The gene arrangement *fliL*, *pflI*, *motE*, *motA*, *motK* is conserved in *Paracoccaceae*, but in *S*. *meliloti* and other *Rhizobiaceae* such as *Agrobacterium*, *Ensifer* and *Shinella*, *motE* is found between *flgI* and *flgH*, which encode the flagellar P and L-ring proteins, respectively.

Analysis of the primary sequence of MotE and MotK revealed the presence of a signal sequence suggesting that these proteins are exported to the periplasm by the general secretion system (Sec). MotE is predicted as a protein of 19.2 kDa (185 residues) that is processed to a 16.3 kDa polypeptide after the removal of the first 29 residues. This protein has a MotE domain (COG 3334), previously characterized only in *S*. *meliloti* (S1 Fig). Structure predictions of this protein revealed a continuous α-helix from residues 56 to 125, prone to form a coiled coil from residues 57 to 115 (https://www.uniprot.org/uniprotkb/Q3IY88/entry).

**Fig 1:**
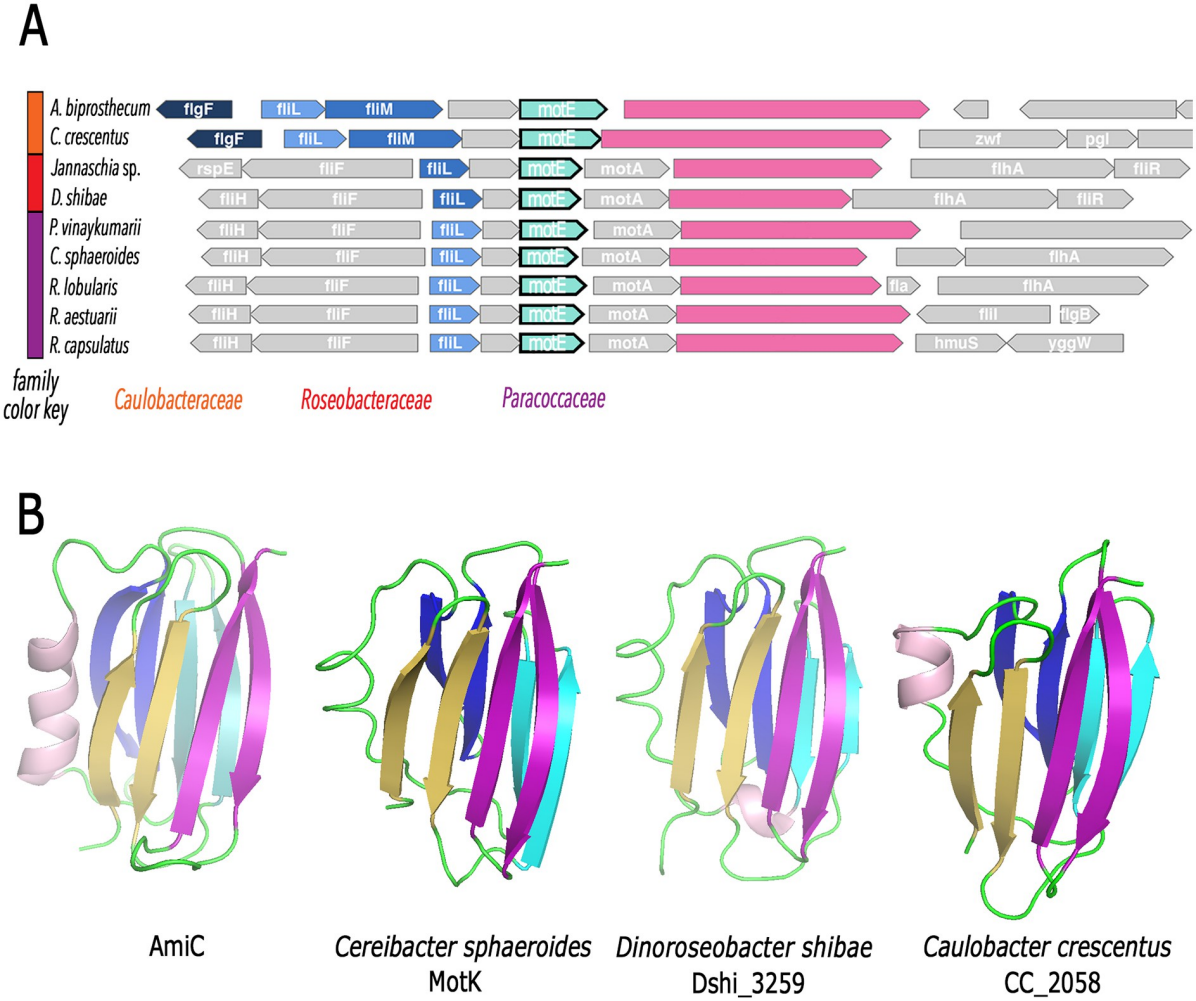
*motE* gene context organization in selected α-proteobacteria and probable AMIN domain in MotK. (A) *motE* (in green) was used as query sequence. Homologues to *motK* (pink) are found in *Paracoccaceae* and in *Roseobacteracea* but with a low degree of similarity. In *Caulobacteracea*, the genes labeled in pink are not similar to *motK* in a BLAST search but the N-terminal region of the products of these genes share structural similarity with MotK. (B) structural model of the N-terminal region of MotK and the proposed homologues in *Dinoroseobacter shibae* (Dshi_3259) and *C*. *crescentus* (CC_2058). These structures correspond to those predicted with alphafold AF-A8LMR7-F1-model_v4) (*D*. *shibae*), AF-Q3IY86-F1-model_v4 (*C*. *sphaeroides*) and AFQ9A6M9-F1-model_v4 (*C*. *crescentus*). At the left, the structure of the N-terminus of AmiC is shown (4bin.pdb). Residues shown are as follows: AmiC 34 to 145, *D*. *shibae* 19 to 117, *C*. *sphaeroides* 23 to 119 and *C*. *crescentus* 36 to 132.

MotK is predicted as a protein of 68.5 kDa (652 residues) that is processed to a 66.3 kDa polypeptide after the removal of the signal peptide (24 residues) (S1 Fig). Structure predictions showed a N-terminal region formed by 8 β-strands (residues 23 to 119) and a short α-helix. Near the C-terminus, a region consisting of α-helices (residues 260–535) was also predicted with a high confidence score. In contrast, the region between these domains and the C-terminus showed a low confidence score and consisted of four disordered regions (residues 92–134, 171–191, 201–220 and 541–608) and two regions with a high proline content (residues 111–127 and 554–570) (https://www.uniprot.org/uniprotkb/Q3IY86/entry). Interestingly, when the N-terminal region (residues 23–120) tertiary structure was modeled using I-Tasser or SWISS-MODEL, the AMIN peptidoglycan binding domain of the AmiC amidase was the best hit, followed by that of PilQ [114, 115]. The structural prediction of residues 121 to 652 of MotK showed similarity with proteins containing tetratricopeptide repeat motifs. A comparative analysis of the *motE* gene cluster organization revealed that in several microorganisms from the *Paracoccaceae*, *Roseobacteracea* and *Caulobacteraceae* families, a large gene (of 2,000 to 3000 bp) encoding a periplasmic protein was present in this region, usually after *motA* or *motE* (Fig 1A and S2 Fig). The tertiary structure of the N-terminal region of these proteins is similar to the AMIN peptidoglycan binding domain (Fig 1B), suggesting that these proteins found in some *Roseobacteracea* species, *Caulobacter crescentus* and other species of *Caulobacteraceae* represent distant homologues of MotK.

### MotE and MotK are required for flagellar rotation

**Fig 2:**
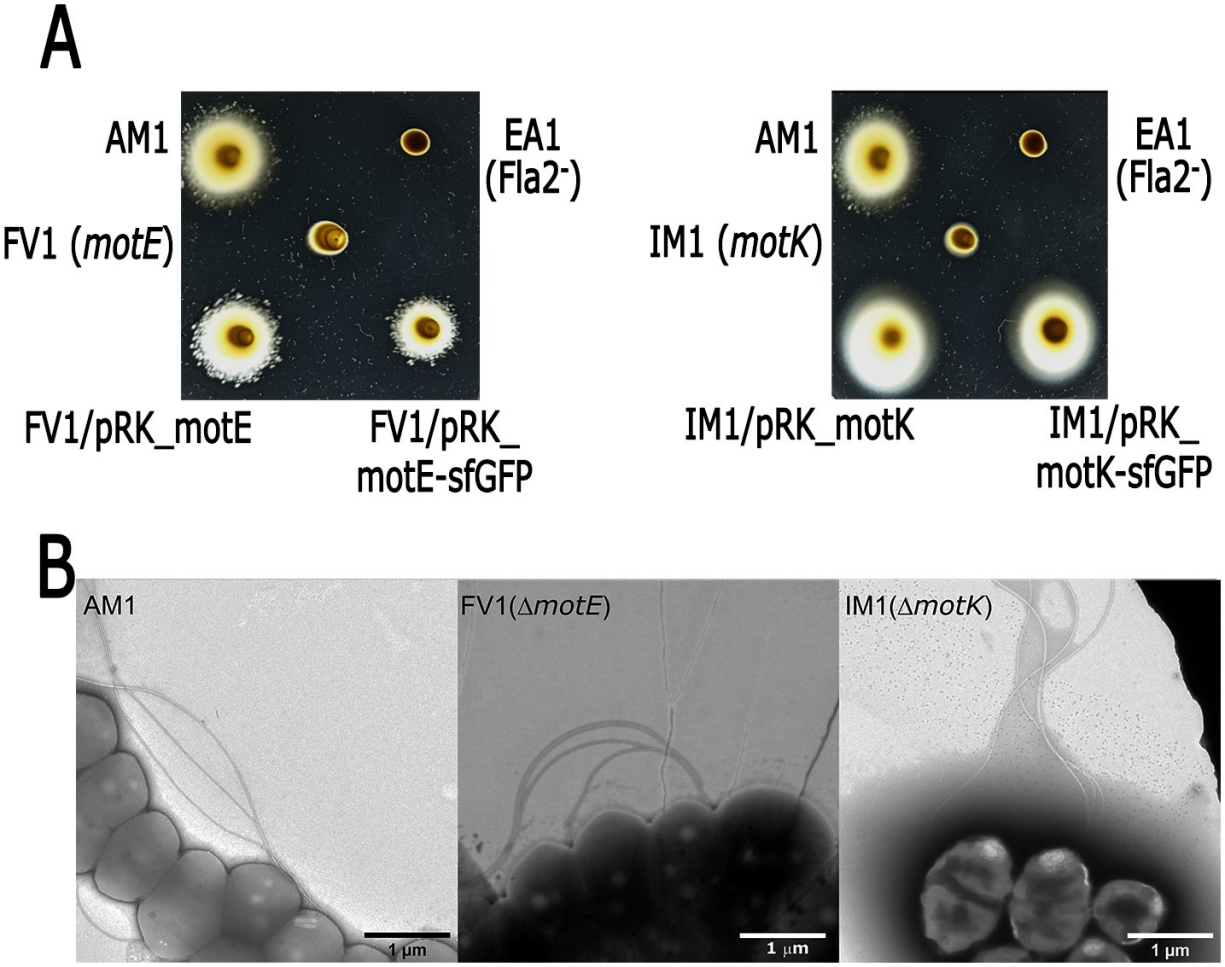
Phenotypic analysis of strains FV1 and IM1. (A) swimming plates. At the left, the plate was inoculated with the parental strain AM1, the Fla2^-^ strain EA1(Δ*ctrA*::*aadA*) as negative control, FV1 (*motE*::*aadA*) and the complemented strains carrying pRK_motE or pRKmotE-sfGFP plasmids. At the right, the plate was inoculated with AM1, EA1, IM1 (*motK*::*aadA*) and the complemented strain carrying pRK_motK or pRK_motK-sfGFP. (B) transmission electron microscopy of strains AM1, FV1 and IM1 showing the presence of flagella in both mutant strains. Scale bars, 1 μm.

Mutants in *motE* and *motK* genes were isolated in order to determine if the product of these genes were required for motility. The AM1 derivatives *motE*::*aadA* and *motK*::*aadA* mutant strains were unable to swim in liquid medium or soft-agar plates (Fig 2A); however, when cells of both mutants were examined by electron microscopy, flagellar filaments were observed (Fig 2B). In agreement with this result, the amount of extracellular hook protein (FlgE2) present in the supernatant of cell cultures subjected to mechanical shearing of flagella was similar to that detected for AM1 (S3 Fig). In consequence, we concluded that these mutants have a paralyzed phenotype. FV1 (*motE*::*aadA*) and IM1 (*motK*::*aadA*) strains regained swimming proficiency when they were complemented with a plasmid expressing *motE* or *motK*, respectively (Fig 2A), indicating that the observed phenotypes are indeed caused by the introduced gene interruptions.

### MotE and MotK are periplasmic proteins

**Fig 3:**
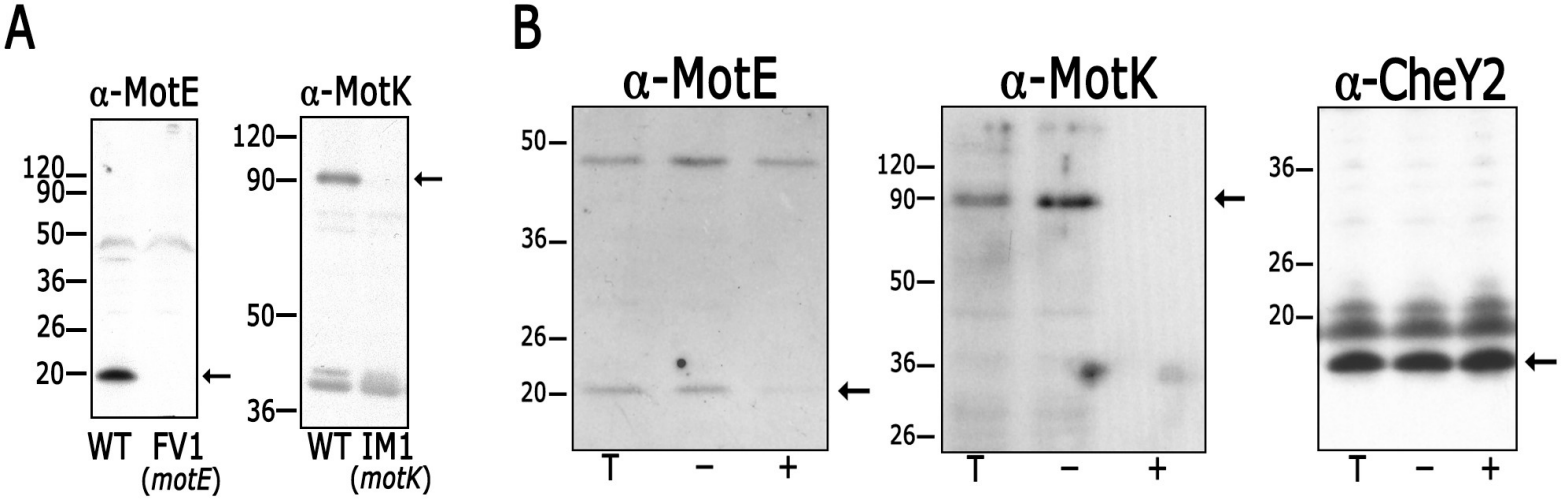
Detection of MotE and MotK by Western blot and protease susceptibility of MotE and MotK in AM1 spheroplasts. (A) Immunoblotting using total cell extracts of AM1 (WT) and the mutant strains FV1 (*motE*::*aadA*) or IM1 (*motK*::*aadA*). Arrows indicate the band corresponding to each polypeptide. Non-specific recognition of other polypeptides commonly occurred; however, these proteins show a different migration, and they are also present in the mutant cell extracts. Migration of the molecular mass markers is shown at the left and values are expressed in kDa. (B) Susceptibility of MotE and MotK to proteinase K tested in spheroplasts of AM1. Spheroplast were incubated for 15 min in the presence (+) or absence of proteinase K (-). Lanes labelled with the letter T, correspond to total cell extracts from AM1. The samples were analyzed by western blot using specific antibodies raised against MotE, MotK or the cytoplasmic protein CheY2. Arrows indicate the bands corresponding to each polypeptide. Similar results were observed when the experiments were performed in at least three independent occasions.

To confirm the periplasmic localization of MotE and MotK, a protease sensitivity assay was carried out using polyclonal antibodies raised against MotE or MotK. These antibodies were tested by Western blot using total cell extracts of strains AM1, FV1 or IM. The Western blot showed a clear band that approximately agrees with the predicted molecular weight of MotE in AM1 sample that is absent in FV1, indicating that this polypeptide corresponds to MotE (Fig 3A left panel). For MotK a conspicuous signal of approximately 90 kDa was detected in AM1 that is absent in IM1, indicating that MotK presents an anomalous migration (Fig 3A, right panel). Once the specificity of the antibodies was validated, we tested the susceptibility to proteinase K of the MotE and MotK proteins in AM1 spheroplasts. As shown in Fig 3B, MotE was clearly detected in total cell extracts and in spheroplasts, but the signal of the protein disappeared after treatment of the spheroplasts with proteinase K. A similar result was obtained when MotK was tested using this protocol. The cytoplasmic CheY2 protein was used as a negative control; in this case, the protein was still visible after treatment of the spheroplasts with proteinase K, confirming both its cytoplasmic localization and the stability of the spheroplasts (Fig 3B).

### MotE interacts with itself through a disulfide bridge

**Fig 4:**
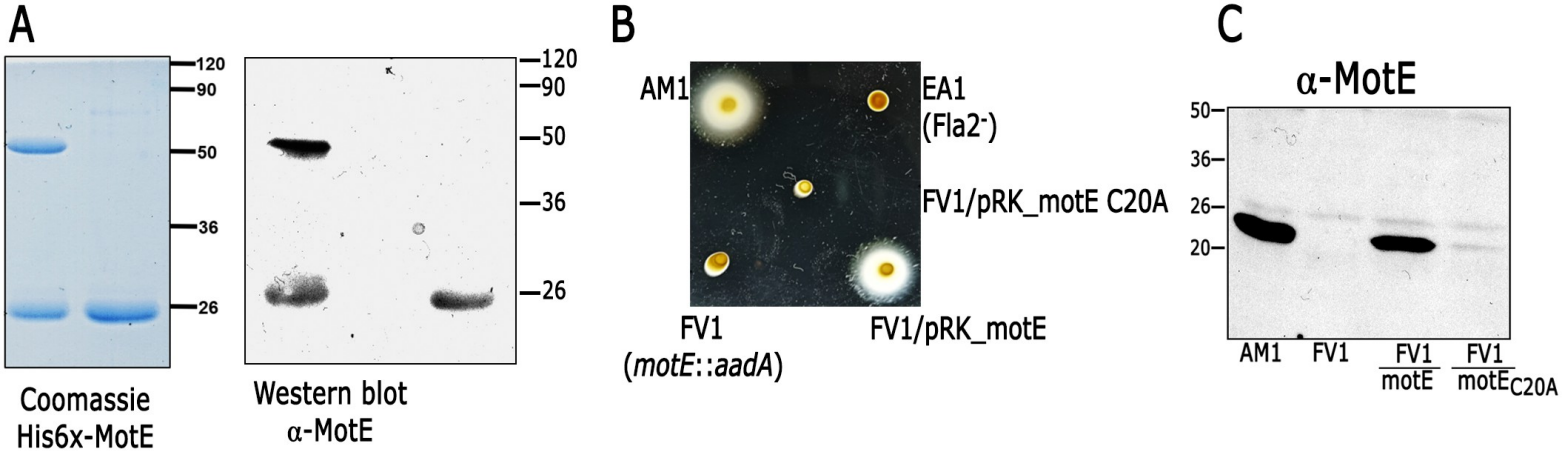
Dimerization of MotE and relevance of the residue Cys20 for the function of MotE. (A) Purified His6x-MotE was subjected to SDS-PAGE and stained with Coomassie blue. Samples were prepared in the absence (left line) or presence (right line) of β-mercaptoethanol in the loading buffer. The image on the right side shows an immunodetection of MotE using total cell extracts of AM1 prepared in the absence (left line) or presence (right line) of β-mercaptoethanol, these samples are separated by an empty lane. (B) Swimming ability of FV1 strain carrying pRK_motE or pRK_motE C20A evaluated in soft agar plates. AM1 and a Fla2^-^ strain (EA1), were used as positive and negative controls, respectively. (C) Total cell extracts of FV1 carrying pRK_motE or pRK_motE C20A were analyzed by immunoblotting using anti-MotE antibody. Similar results were observed when the experiments were performed in three independent occasions.

During the purification procedures of His6x-MotE we often detected the presence of a band of ca. 40 kDa that is approximately the size of a dimer of His6x-MotE (19.9 kDa). It was considered that a single cysteine (C) residue at position 20 of the mature protein could be forming a disulfide bridge between monomers. We confirmed that in the absence of β-mercaptoethanol, the 40 kDa polypeptide was clearly detected whereas the monomeric form is dominant in the presence of freshly added β-mercaptoethanol. A similar result was observed when a total cell extract of AM1 was analyzed by Western blot in the presence or absence of this reductor agent (Fig 4A). This suggests that MotE is a dimer in the periplasm of the cell. To test the relevance of the dimeric form of MotE, we replaced the cysteine residue by alanine and expressed it in strain FV1 (*motE*::*aadA*). We noticed that this variant of the protein does not complement the paralyzed phenotype of FV1 (Fig 4B). However, MotE C20A was undetectable in total cell extracts of FV1/pRK_motE C20A, indicating that this substitution makes the protein unstable (Fig 4B). This result prevented us from determining if the dimeric form is essential for MotE function; however, a similar observation was reported for the mutant version MotE C53A of *S*. *meliloti* [83], suggesting that the intrinsic stability of the protein in the periplasm must be dependent on the disulfide bridge formed by this residue and that MotE is present at least as a dimer.

### MotE, MotK and MotB2 interact between them

**Fig 5:**
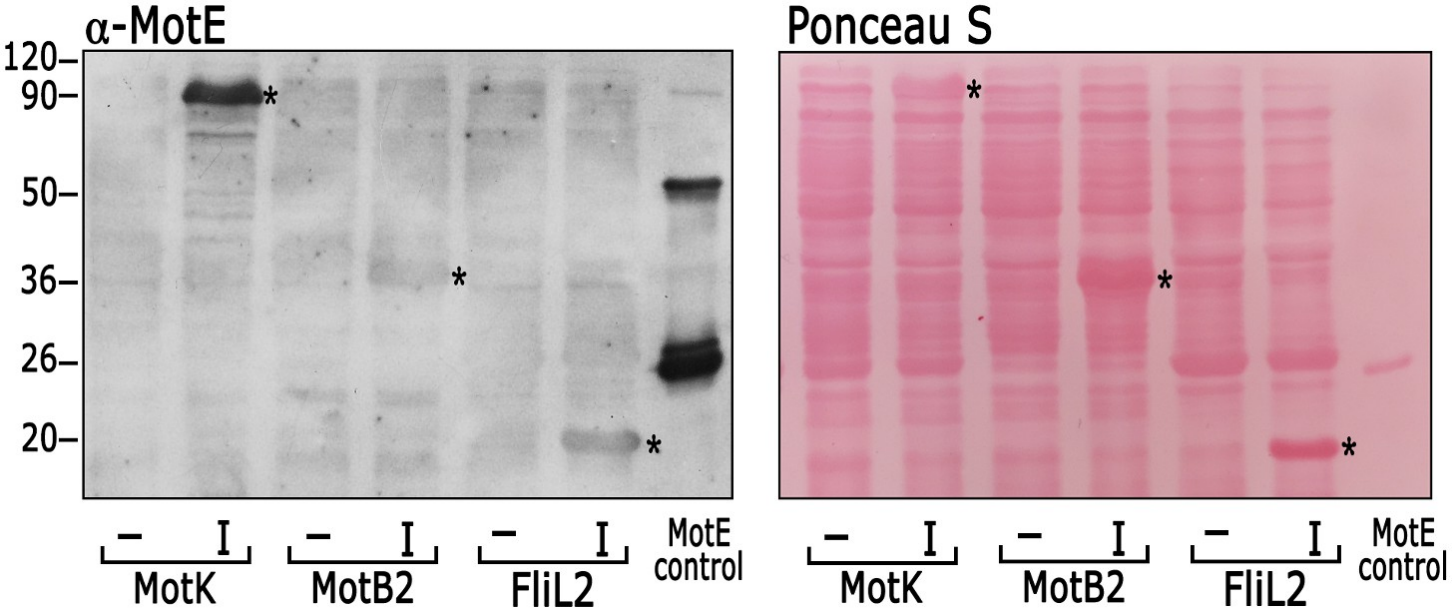
Far Western blot testing the possible interactions of MotE with MotK, MotB2 and FliL2. *E*. *coli* cell extracts induced (I) or not induced (-) for the expression of MotK, MotB2 or FliL2 were subject to SDS-PAGE, transferred to nitrocellulose and incubated with 5 μg of soluble His6x-MotE for 4h. The membrane was washed, and the presence of MotE was detected using anti-MotE antibodies. An asterisk denotes the band corresponding to MotK, MotB2 or FliL2. His6x-MotE was loaded as a positive control. At the right of the figure, the membrane stained with Ponceau S is shown. The experiment was performed at least three times, a representative image is shown.

To shed some light on the role of MotE and MotK, we tested if MotE could interact with other components of the flagellar motor. The interaction of MotE with MotK, MotB2, and FliL2 was tested by Far Western, using total cell extracts of *E*. *coli* expressing MotK, MotB2 and FliL2, and the soluble His6x-MotE protein. As shown in Fig 5, MotE was able to interact with MotK. However, it was not possible to conclude if MotE interacts with MotB2 or FliL2, given the weak and ambiguous signal detected.

**Fig 6:**
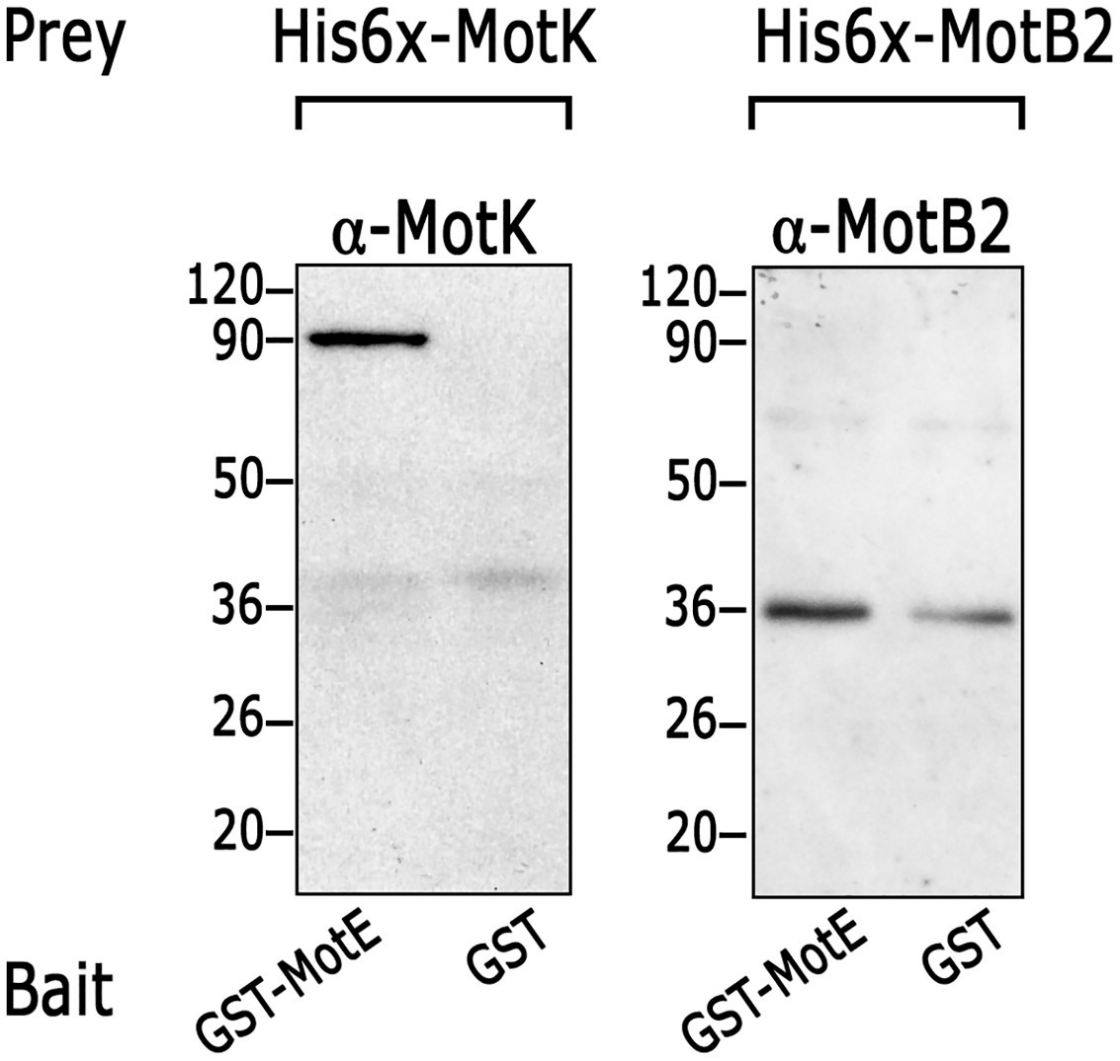
Pulldown of MotK and MotB2 with MotE. Pulldown assays of purified His6x-MotK and His6x-MotB2 with GST-MotE and GST alone were carried out and the eluates were analyzed by Western blot. The samples were tested using the appropriate antibody, *i*.*e*. anti-MotK or anti-MotB2. Similar results were observed when the experiments were performed in three independent occasions.

The interaction of MotE with these proteins was also tested in a pull-down experiment utilizing GST-MotE as a bait and His6x-MotK or His6x-MotB2 as pray. This assay confirmed the interaction of MotE with MotK, and with MotB2, although in this case a weak interaction between MotB2 and GST was observed (Fig 6). From these experiments, we conclude that MotE interacts with MotK and with MotB2.

**Fig 7:**
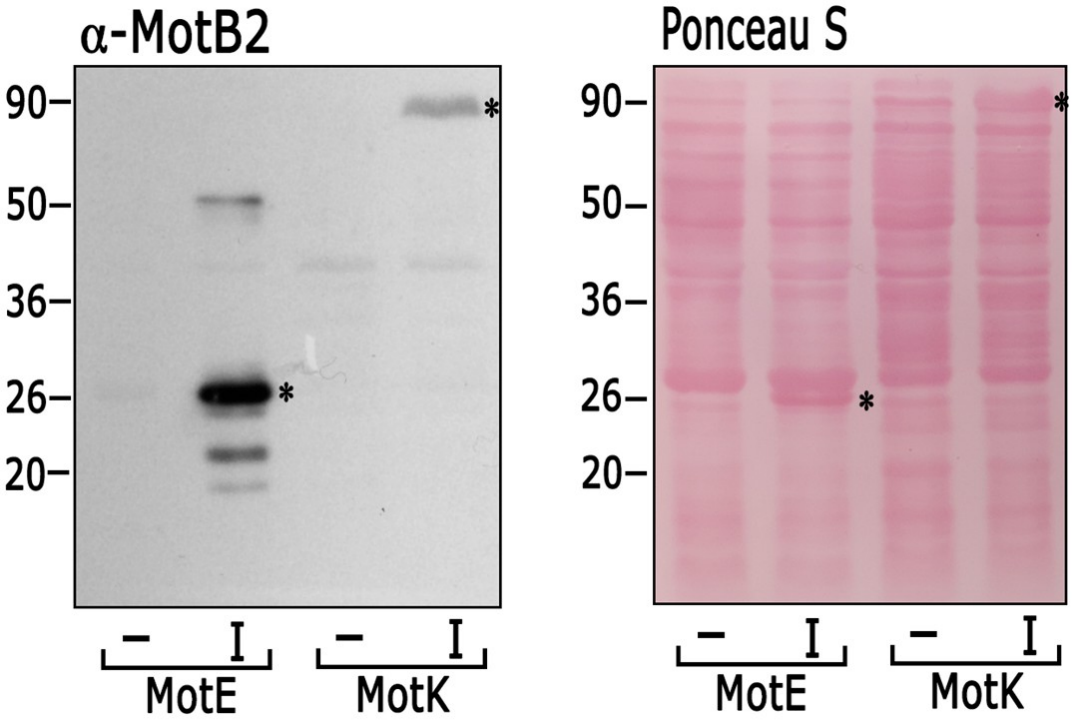
Far Western blot testing the possible interactions of MotB2 with MotE and MotK. *E*. *coli* cell extracts induced (I) or not induced (-) for the expression of MotE and MotK were subjected to SDS-PAGE transferred to nitrocellulose and incubated with 5 μg of His6x-MotB2 for 4 h. After washing, the presence of MotB2 was detected using anti-MotB2 antibodies. An asterisk denotes the band corresponding to MotE and MotK. At the right of the figure, the membrane stained with Ponceau S is shown. The experiment was performed at least three times, a representative image is shown.

To further test the interaction between MotE and MotB2 and to determine if MotK also interacts with MotB2, the interaction between these proteins was also probed in a Far Western assay using MotB2 as a soluble protein. A strong interaction between MotB2 and MotE was observed and a weaker but clear interaction between MotK and MotB2 was detected (Fig 7). These results confirmed the interaction of MotE with MotB2 and provide evidence of an interaction between MotK and MotB2.

### MotE stability depends on MotK but not vice versa

**Fig 8:**
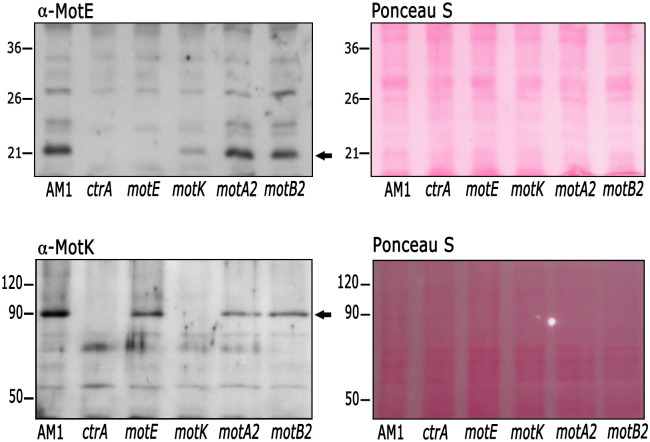
Stability of MotE and MotK proteins in different genetic backgrounds. The presence of MotE and MotK in the absence of other flagellar components was evaluated by Western blot using total cell extracts of the indicated mutant strains and anti-MotE or anti-MotK antibodies. An arrow indicates the band corresponding to MotE or MotK. At the right of the figure, the membranes stained with Ponceau S are shown. Similar results were observed when the experiments were performed in three independent occasions.

In the periplasm, formation of protein complexes in many cases stabilizes the proteins involved; for this reason, we determined if MotE is stable in the absence of other flagellar motor proteins. Fig 8 shows that MotE was detected in total cell extracts obtained from *motA2* and *motB2* strains, but it was severely reduced in the sample obtained from the *motK* mutant strain. In agreement with previous reports indicating that CtrA is required to express the flagellar *fla2* genes, MotE was not detected in the absence of this transcriptional activator (Fig 8). In contrast, MotK was detected in the absence of MotE, MotA2 and MotB2; similarly to MotE, MotK is absent in the Δ*ctrA* strain (Fig 8). In some assays the MotE and MotK signal was reduced in the *motA2* and *motB2* mutant strains as compared with AM1; however, these results were variable and a more quantitative approach will be needed to clarify this matter. We also examined the stability of MotB2 in the *motE* and *motK* mutants and we did not observe an evident change (S4 Fig).

### MotE and MotK localize to the flagellated cell pole

**Fig 9:**
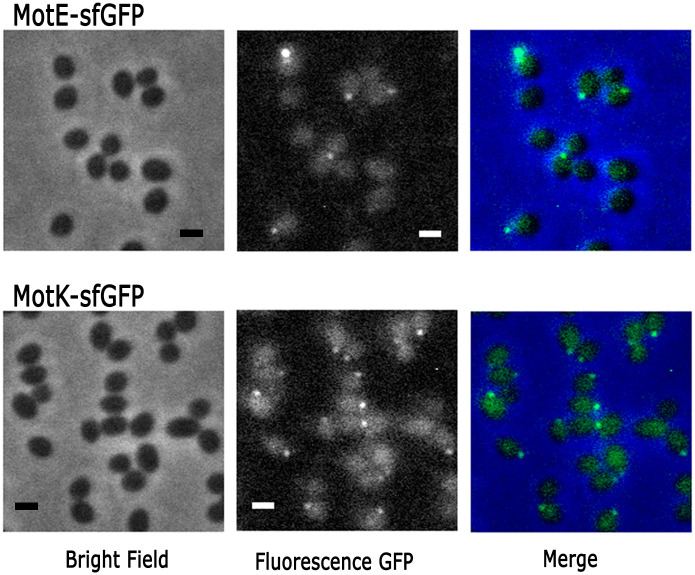
MotE-sfGPF and MoK-sfGFP detection by fluorescence microscopy. Strains FV1 and IM1 carrying pRK_motE-sfGFP or pRK_motK-sfGFP were grown anaerobically in 0.2% casamino acids at a OD_600_ of 0.6, and promptly observed by Phase contrast microscopy (Bright field) or by fluorescence microscopy. Scale bars, 1 μm.

**Fig 10:**
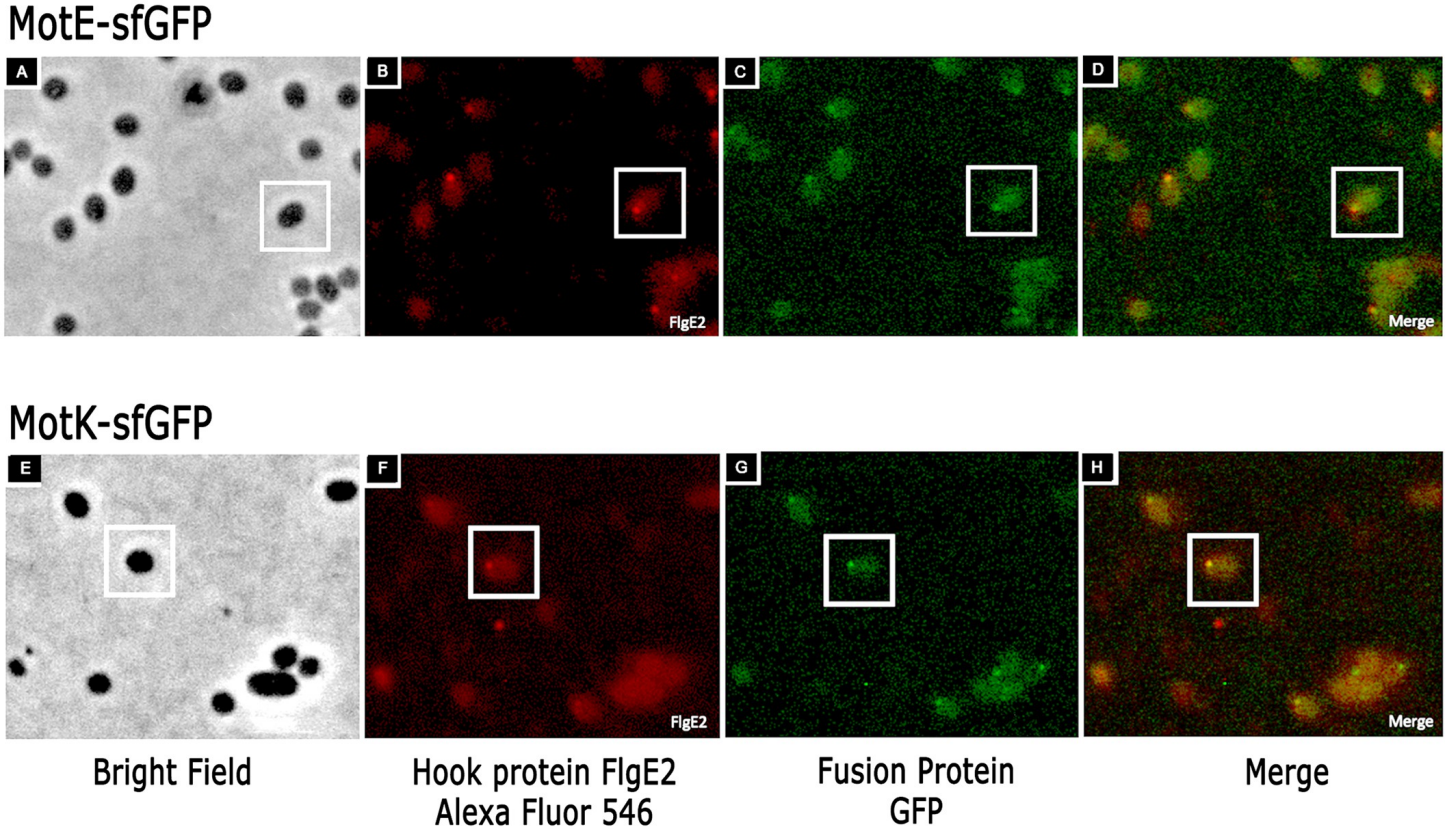
Colocalization of MotE-sfGFP and MotK-sfGFP with the bacterial hook. Detection of the flagellar hook protein FlgE2 was done by using anti-FlgE2 labeled with Zenon Alexa Fluor 546. Images in A, B, C and D correspond to FV1/pRK_MotE-sfGFP: observed by phase contrast microscopy in A, or fluorescence microscopy, B and C. The merge image from B and C is shown in D. Images labeled as E, F, G and H correspond to IM1/pRK_MotK-sfGFP and follow the same order than the images of MotE-sfGFP. A white square denotes the same cell across A to D or E to H.

Our results suggest that MotE and MotK could be part of the flagellar structure and therefore, they should be localized at the flagellar pole. To test this prediction, the fusion proteins MotE-sfGFP and MotK-sfGFP were constructed and cloned in the expression vector pRK415. These fusion proteins were able to complement the swimming phenotype of the mutant strains FV1 and IM1, respectively (Fig 2), indicating that they are functional. Fluorescence microscopy of FV1 cells expressing MotE-sfGFP revealed that approximately 42% of the cells showed a single fluorescent focus at the cellular pole (Fig 9). Similarly, MotK-sfGFP was expressed in FV2 strain (Δ*motK2*::hyg), and approximately 52% of the cells showed a single fluorescent focus at the cell pole (Fig 9). The presence of a fluorescent focus generated by either MotE-sfGFP or MotK-sfGFP in approximately a 50% of the population correlates with the number of motile cells usually present in the culture. In agreement with the idea that MotE and MotK are part of the flagellar structure, we observed that the focus of MotE-sfGFP and MotK-sfGFP colocalizes with the flagellar hook detected by immunocytochemistry (Fig 10).

**Fig 11:**
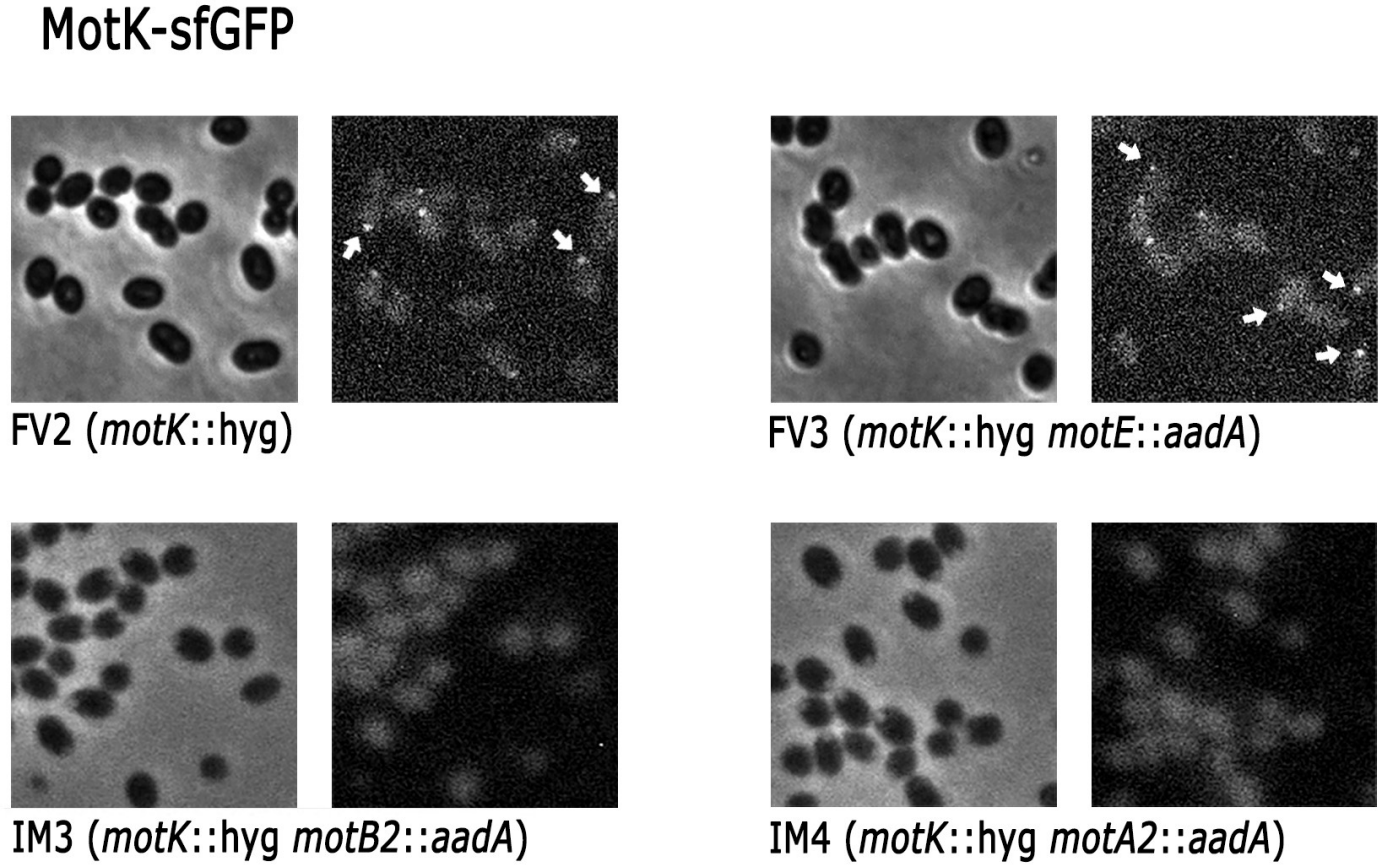
MotK-sfGFP detection in different strains. Strains FV2, FV3, IM3 and IM4 carrying pRK_motK-sfGFP were grown anaerobically in 0.2% casamino acids at a OD_600_ of 0.6 and observed by phase contrast (left) or fluorescence microscopy (right). Representative fluorescent foci are indicated with white arrows.

**Fig 12:**
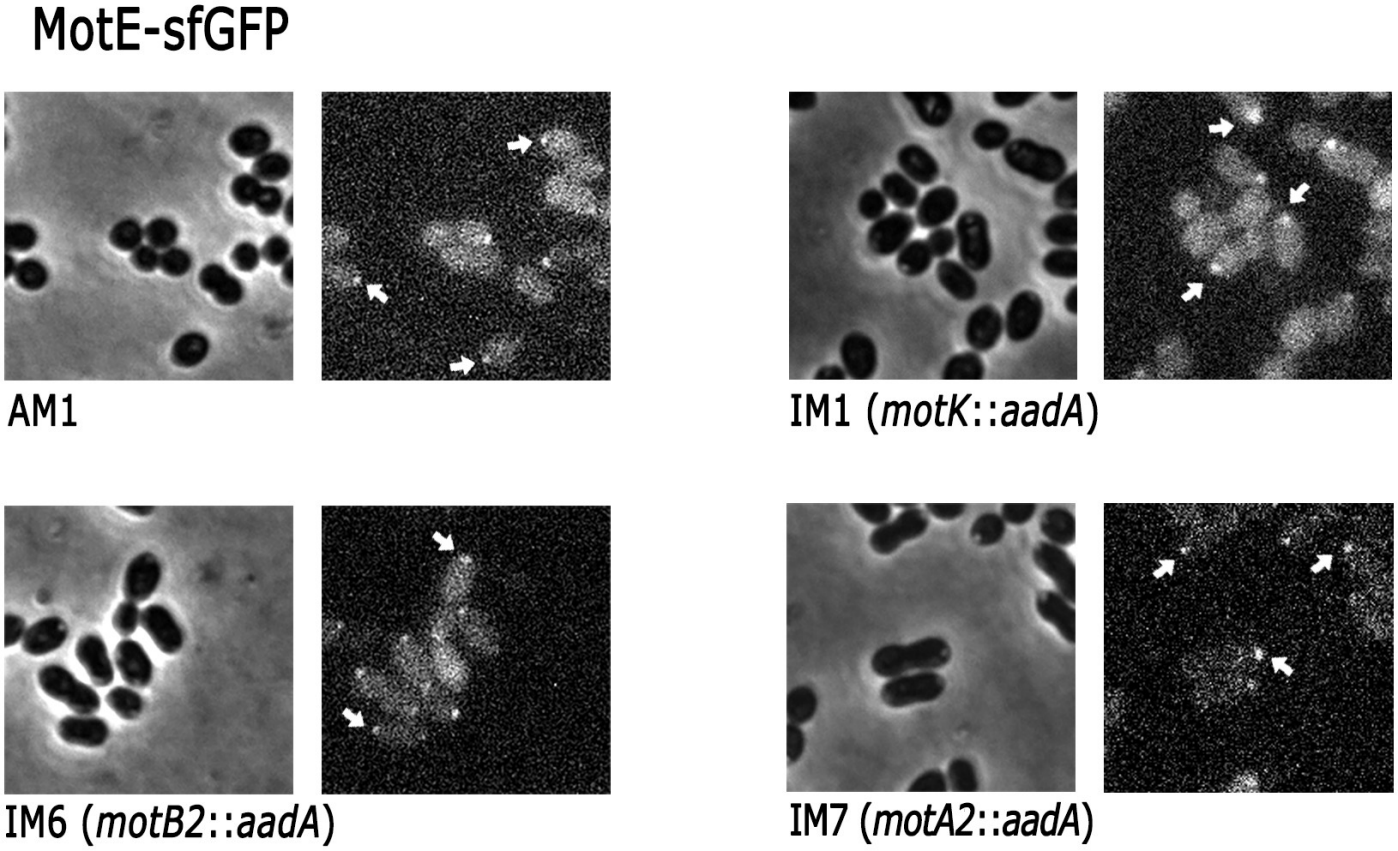
MotE-sfGFP detection in different strains. Strains AM1, IM1, IM6 and IM7 carrying pRK_motE-sfGFP were grown anaerobically in 0.2% casamino acids at a OD_600_ of 0.6 and observed by phase contrast (left) or fluorescence microscopy (right). Representative fluorescent foci are indicated with white arrows.

**Fig 13:**
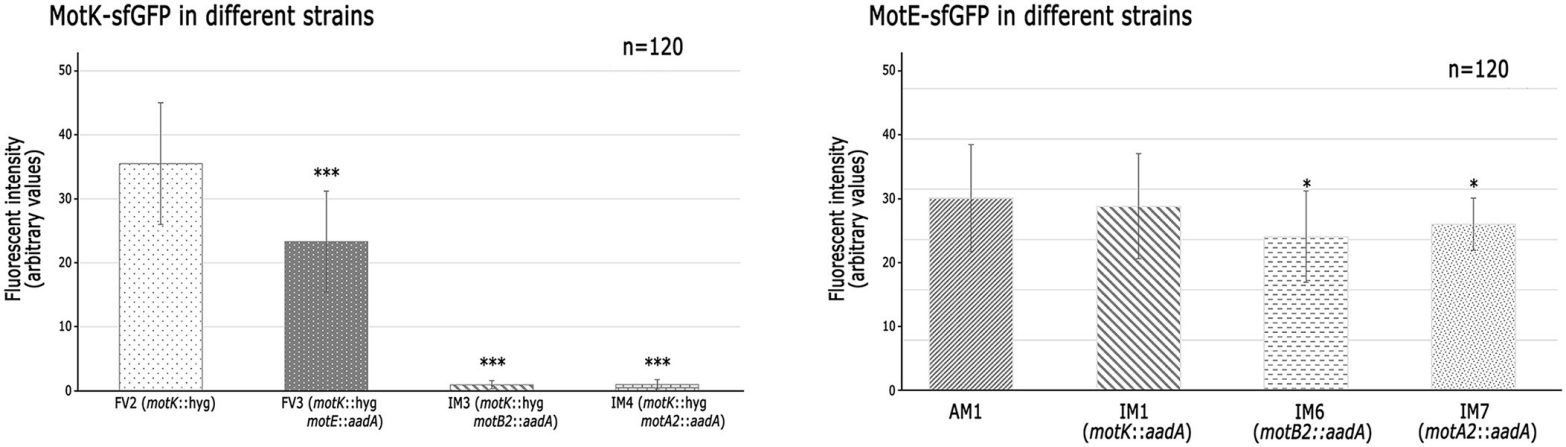
Fluorescence intensity of MotK-sfGFP and MotE-sfGFP foci across different strains. The graphs show the average intensity of the foci of the two fluorescent fusions in different stains. Significance against FV2 or AM1 is indicated; *** P ≤ 0.001, * P ≤ 0.05. Fluorescence intensities and the statistical analysis were determined as described in Materials and Methods.

Given that MotE-sfGFP and MotK-sfGFP are transcribed from the *lac* promoter present in pRK415, and in consequence their expression is independent of the flagellar regulon, we evaluate if their localization was affected in the absence of the stator proteins MotA2 and MotB2. As shown in Fig 11, MotK-sfGFP is localized in the absence of MotE but not in the absence of MotB2 and MotA2 proteins (Fig 11). In contrast, MotE-sfGFP was localized in the absence of MotA2, MotB2 and even in the absence of MotK (Fig 12). This last result was surprising since our previous results showed that MotE was degraded in the absence of MotK, we presume that the sfGFP moiety protects and stabilizes MotE. Fluorescence intensity of the focus generated by MotE-sfGFP and MotK-sfGFP was quantified for 120 independent cells of each strain. This analysis corroborated that MotK-sfGFP is not localized in the absence of MotB2 and MotA2 and reveled a reduction of the intensity of the fluorescent foci in the absence of MotE (Fig 13). Fluorescence intensity of MotE-sfGFP did not show a significant change in the absence of MotK, but in the absence of MotB2 and MotA2, a slight reduction was detected (Fig 13). These results revealed the relevance of the interactions of MotK and MotE with the flagellar stator proteins, MotA2 and MotB2.

## Discussion

**Fig 14:**
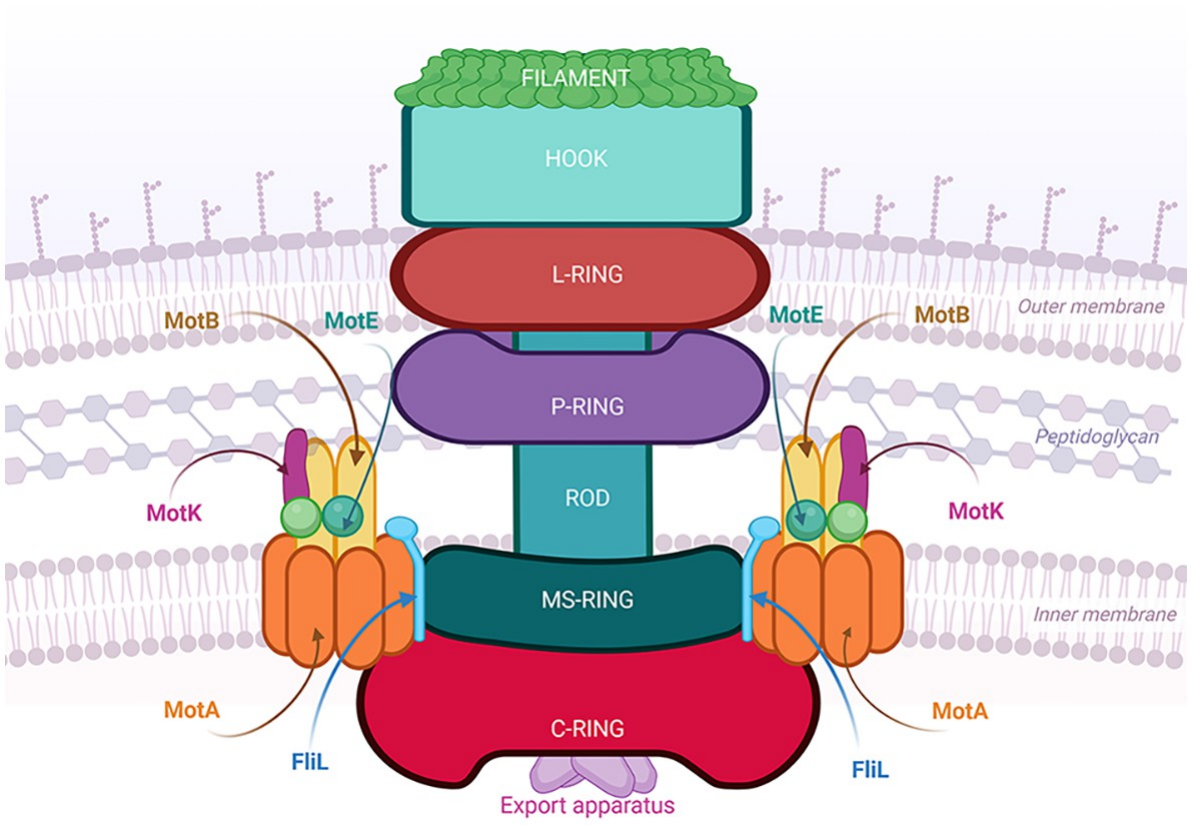
Model showing the localization of the periplasmic proteins MotK and MotE in the flagellar motor. The schematic represents the possible interaction of MotK with the peptidoglycan wall, and with the MotB and MotE proteins observed in this work. Likewise, the possible interactions of MotE with MotB and MotK are represented.

Recently, observation of the flagellar motor by cryoET and cryoEM in different bacterial species, revealed that this structure is very diverse and more complex than previously described for *E*. *coli* and *S*. *enterica* [1, 46, 48, 63–65]. The characterization of these new structures and their components not only will contribute to describe the flagellar motor in different species but also will allow us to understand how the accessory proteins modify the essential features of the motor to adjust it to particular needs. In this context, in this work we demonstrated that rotation of the Fla2 flagella of *C*. *sphaeroides* depends on MotE and the novel MotK protein. We established the periplasmic localization of both proteins and determined that MotK is stable in the absence of other flagellar components involved in rotation such as MotA2, MotB2 and MotE. Likewise, MotE is stable in the absence of MotA2 and MotB2 but not in the absence of MotK. This supports our *in vitro* results that indicate that MotE interacts with MotK and suggests that these proteins probably form a stable complex in the periplasm. We also show evidence suggesting that MotK and MotE could be part of the flagellar structure since they localize to the flagellated pole. The Mot- phenotype of the mutants suggests that these proteins promote the recruitment of MotB2 to the flagellar motor or are necessary to remodel the proton channel during activation. Any of these possibilities agrees with the fact that MotK and MotE interact *in vitro* with MotB2, and that *in vivo* MotK localization is dependent on MotB2 and MotA2, and partially on MotE. Likewise, recruitment of MotE to the flagellar motor seems to be reduced in the absence of MotA2 and MotB2. These results suggest that MotK and MotE are part of the flagellar stator (Fig 14), and this could explain why these proteins were not previously identified in the isolated flagellar structures, since that the stator complexes are usually lost during basal body purification. In agreement with this idea, the N-terminal domain of MotK may bind peptidoglycan since it shows structural similarity to the AMIN domain. This would allow MotK to anchor MotE and to favor the open conformation of MotB. In this regard, analysis of the gene cluster organization of *motE*, revealed what could be considered distant homologues of MotK in several microorganisms, characterized by the presence of a N-terminal region with a tertiary structure similar to the peptidoglycan binding domain of AmiC. Interestingly, in the case of *C*. *crescentus* it has been reported that a mutant strain of this gene, CC_2058, shows a Mot^-^ phenotype [116].

It has been previously shown that other flagellar proteins besides the canonical stator and rotor proteins are involved in motor assembly and function by interacting or remodeling the stator complexes. In bacteria, the most prevalent of these proteins is the membrane protein FliL [53–57, 117]. This monotopic membrane protein has a short cytoplasmatic N-terminus, and a C-terminus localized in the periplasmic space. FliL forms rings around the MotA/MotB complexes [56] and probably contributes to the opening of the proton channel. In this work, we did not detect a positive interaction of MotE or MotK with the periplasmic region of FliL2. However, it is possible that *in vivo* these proteins adopt a conformation that allows these interactions. Alternatively, MotE and MotK could be distally positioned from the periplasmic side of the inner membrane, and only interact with a distal portion of MotB2.

Other flagellar proteins that interact with the stator complexes are the *Vibrio*-specific proteins MotX/MotY that together form a periplasmic ring (T-ring) required to assemble/recruit the stator complexes around the flagellar motor. Biochemical evidence indicates that MotX interacts with PomB, and it has been proposed that this interaction contributes to achieve the high rotational rates observed for this flagellum [58]. In addition, images from cryo-EM of the flagellar motor of *Campylobacter jejuni*, has revealed the existence of several periplasmic rings or disks, the proximal ring, formed by the PflB protein, is essential for recruitment of the stator complexes [46]. In this context, the proteins MotE and MotK could be part of a periplasmic structure required to recruit or stabilize the stators, like the T-ring of *Vibrio* or the basal disk of *C*. *jejuni*.

As previously suggested, the existence of periplasmic rings to reinforce the flagellar motor has emerged several times during evolution since these proteins are not homologues [1, 118]; nevertheless, they likely have a similar function. Once these proteins were recruited to the flagellar system, coevolution of these components with the main components of the flagellar motor made them an essential part of the motor. In this regard, it is possible that MotE has been inherited by *S*. *meliloti* and *C*. *sphaeroides* from an ancestral bacterium, but it adapted to interact with different species-specific periplasmic proteins, *i*.*e*. MotC in *S*. *meliloti* and MotK in *C*. *sphaeroides*. In this process, MotE acquired particular characteristics such as its stability dependency, which is different between these organisms, but likely other features. Characterization of homologues proteins in other bacterial systems will contribute to understand the conserved role of these proteins in the bacterial flagellum.

## Supporting information

S1 Fig Primary sequence and in silico analysis of MotE and MotK. The primary sequence of MotE and MotK are showed. A vertical arrow indicates the putative cleavage site of the signal peptide of MotE and MotK. The domain detected by CD-BLAST present in MotE is shown, as well as the MotK and AmiC alignment obtained with SWISS-MODEL. The arrows represent β-strands and alpha-helixes are rounded squares.
(TIF)

S2 Fig *motE* gene cluster organization in several α-proteobacteria. *motE* homologues are shown in green (COG 3334). In *Rhodobacterales* and *Caulobacterales* this gene is found in the *fliL* operon usually upstream *motA*. In *Sinorhizobium* and *Ensifer* group, *motE* is found between *flgI* and *flgH* (brown). In some species, downstream of *motE* or *motA*, a large gene labeled in pink (*motK* homologues) or purple, encodes for a periplasmic protein with a N-terminal region that predicts a tertiary structure similar to that of the peptidoglycan binding domain of AmiC. When compared to MotK from *C*. *sphaeroides*, the proteins from *Rhodobacterales* (*Paracoccaceae* and *Roseobacteraceae*) species show low similarity, and for this reason are labeled with the same color (pink). However, no similarity was found with the proteins identified in *Caulobacteraceae* (*Caulobacter vibroides*, *Asticcacaulis biprosthecium* and others) labeled in violet.
(TIF)

S3 Fig Detection of FlgE2 and CckA by Western blot in total cell extracts (whole cells) and supernatants cultures after mechanical shearing of the flagella (supernatant). The presence of the hook protein (FlgE2) is observed in the supernatant of all the strains except for the *ctrA* mutant that was used as a negative control since the expression of *flgE2* is dependent on the transcriptional factor CtrA. To verify the integrity of the cells, the samples were also tested with the α-CckA antibody that recognizes an intracellular protein. Arrows indicate the band corresponding to each polypeptide.
(TIF)

S4 Fig Presence of MotB2 in FV1 and IM1 strains. The presence of MotB2 was evaluated in total cell extracts of strains FV1 (*motE*::*aadA*) and IM1 (*motK*::*aadA*) by Western blot. AM1 and IM6 (*motB2*::*aadA*) were included as positive and negative controls, respectively.
(TIF)

S1 Table Oligonucleotides used in this work. (XLSX)

S1 Raw images (PDF)
