# Supplementary figures and images for: Rotation of the Fla2 flagella of Cereibacter sphaeroides requires the periplasmic proteins MotK and MotE that interact with the flagellar stator protein MotB2
Source: PLoS One. 2024 Mar 20;19(3):e0298028. doi: 10.1371/journal.pone.0298028 (PMC10954123; doi:10.1371/journal.pone.0298028)

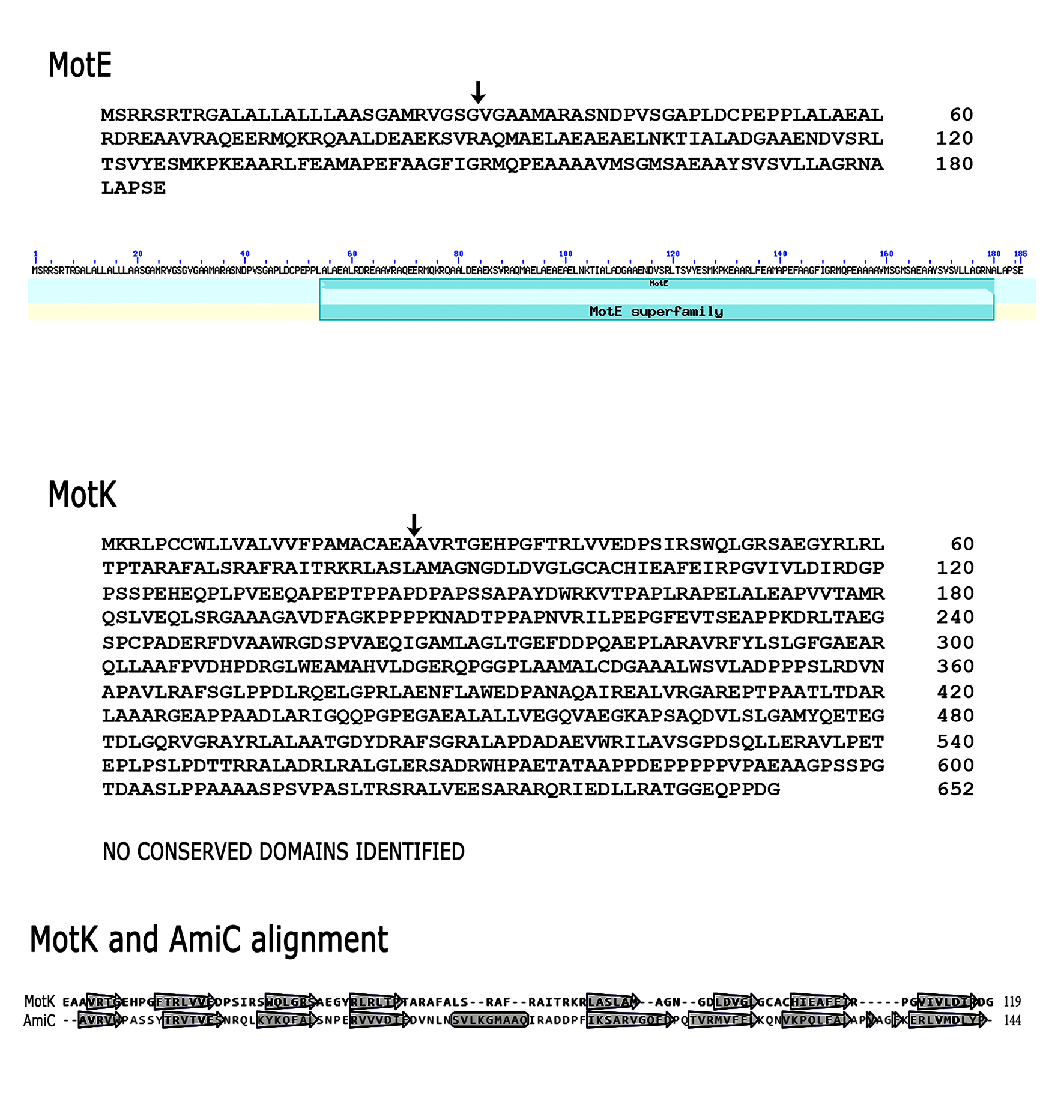

Supplement: S1 Fig — The primary sequence of MotE and MotK are showed. A vertical arrow indicates the putative cleavage site of the signal peptide of MotE and MotK. The domain detected by CD-BLAST present in MotE is shown, as well as the MotK and AmiC alignment obtained with SWISS-MODEL. The arrows represent β-strands and alpha-helixes are rounded squares. (TIF) [file pone.0298028.s001.tif]

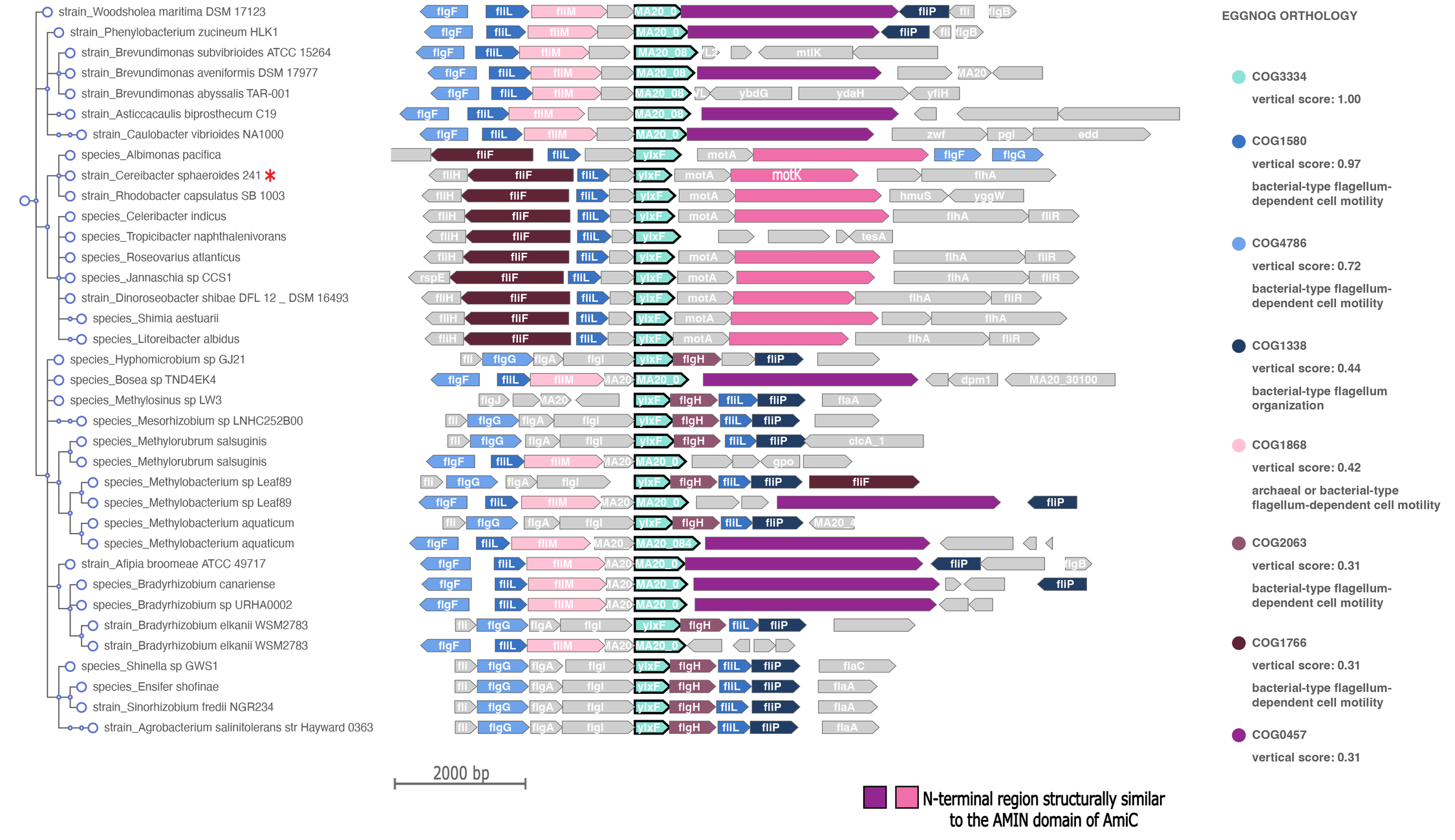

Supplement: S2 Fig — motE homologues are shown in green (COG 3334). In Rhodobacterales and Caulobacterales this gene is found in the fliL operon usually upstream motA. In Sinorhizobium and Ensifer group, motE is found between flgI and flgH (brown). In some species, downstream of motE or motA, a large gene labeled in pink (motK homologues) or purple, encodes for a periplasmic protein with a N-terminal region that predicts a tertiary structure similar to that of the peptidoglycan binding domain of AmiC. When compared to MotK from C. sphaeroides, the proteins from Rhodobacterales (Paracoccaceae and Roseobacteraceae) species show low similarity, and for this reason are labeled with the same color (pink). However, no similarity was found with the proteins identified in Caulobacteraceae (Caulobacter vibroides, Asticcacaulis biprosthecium and others) labeled in violet. (TIF) [file pone.0298028.s002.tif]

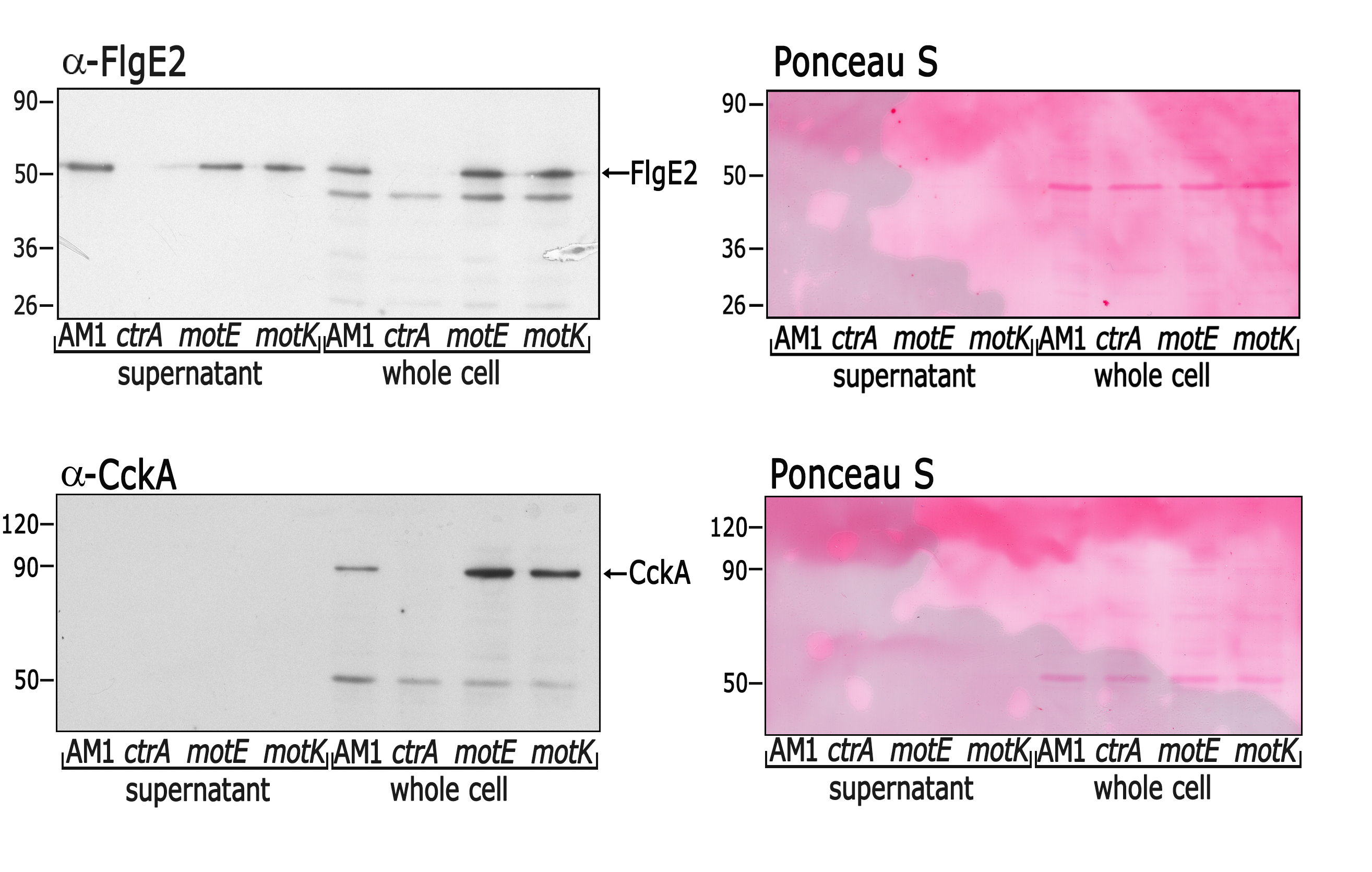

Supplement: S3 Fig — The presence of the hook protein (FlgE2) is observed in the supernatant of all the strains except for the ctrA mutant that was used as a negative control since the expression of flgE2 is dependent on the transcriptional factor CtrA. To verify the integrity of the cells, the samples were also tested with the α-CckA antibody that recognizes an intracellular protein. Arrows indicate the band corresponding to each polypeptide. (TIF) [file pone.0298028.s003.tif]

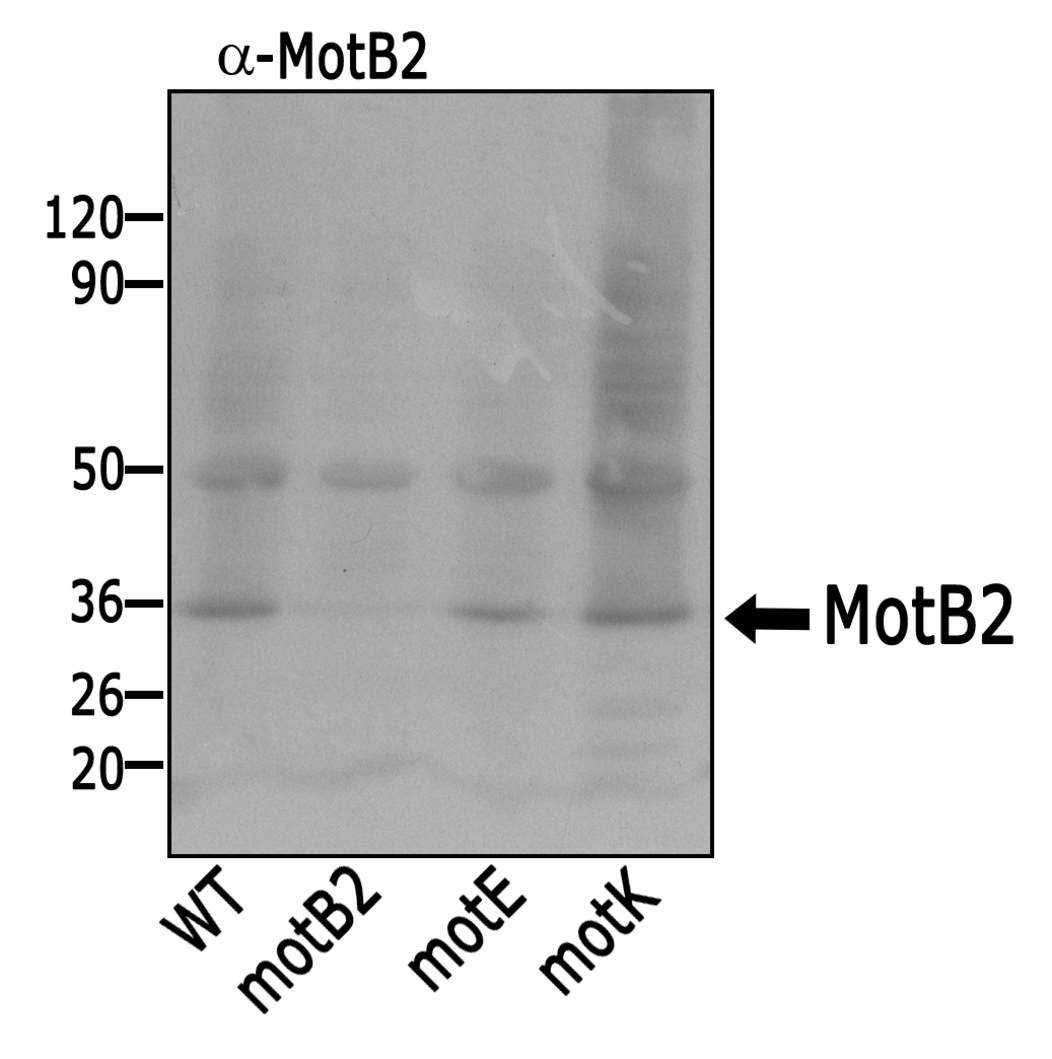

Supplement: S4 Fig — The presence of MotB2 was evaluated in total cell extracts of strains FV1 (motE::aadA) and IM1 (motK::aadA) by Western blot. AM1 and IM6 (motB2::aadA) were included as positive and negative controls, respectively. (TIF) [file pone.0298028.s004.tif]
