# Supplementary material for: Rotation of the Fla2 flagella of Cereibacter sphaeroides requires the periplasmic proteins MotK and MotE that interact with the flagellar stator protein MotB2
Source: PLoS One. 2024 Mar 20;19(3):e0298028. doi: 10.1371/journal.pone.0298028 (PMC10954123; doi:10.1371/journal.pone.0298028)
Supplement: S1 Raw images — (PDF) [file pone.0298028.s006.pdf]

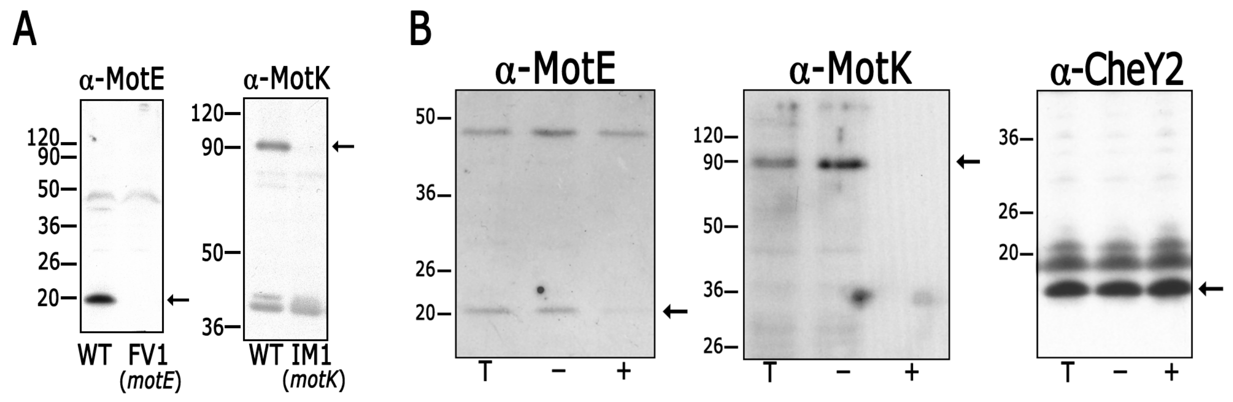

FIG 3

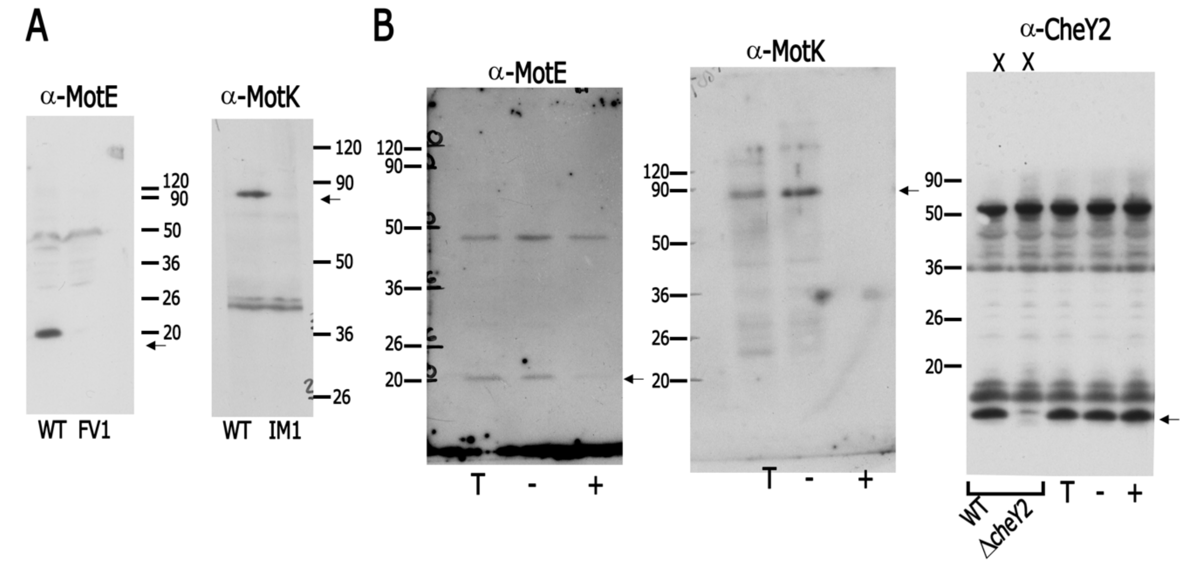

FIG 3 RAW. Scans of X-ray films.

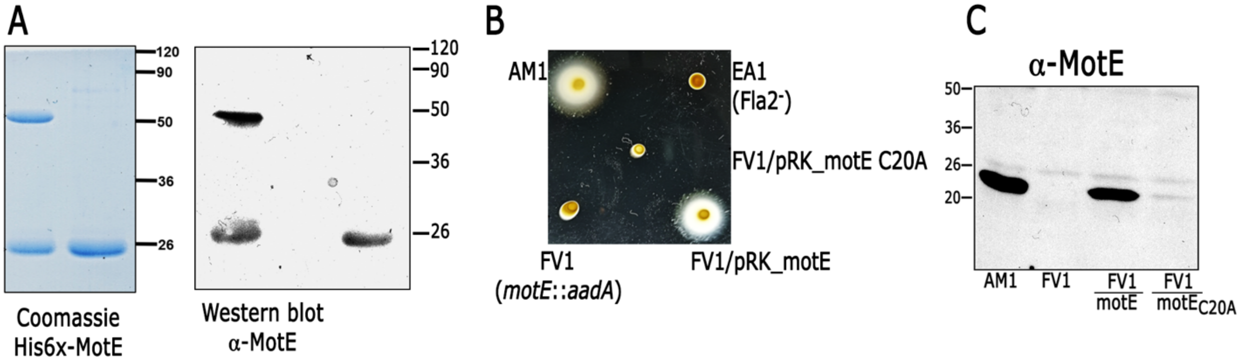

FIG 4

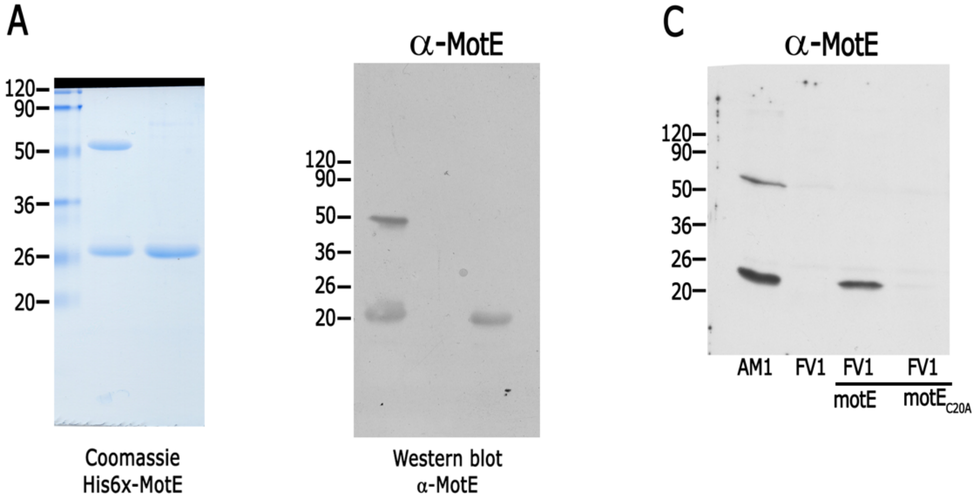

FIG 4 RAW. The image labeled as Coomassie is a digital picture of an SDS-PAGE gel. The images labeled as α-MotE are scans of X-ray films.

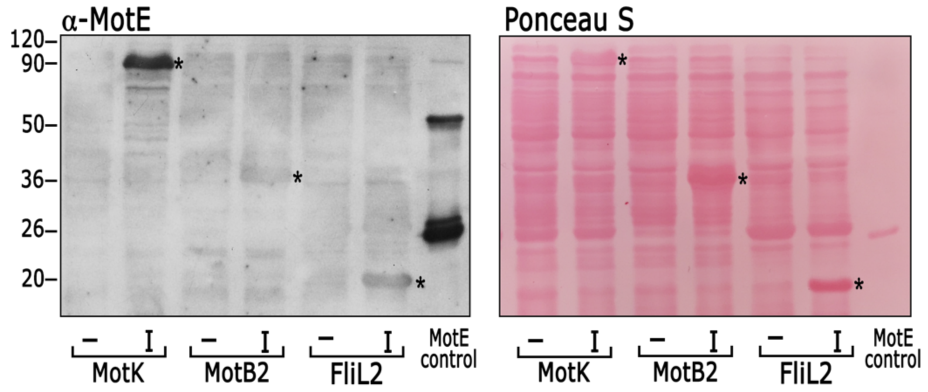

FIG 5

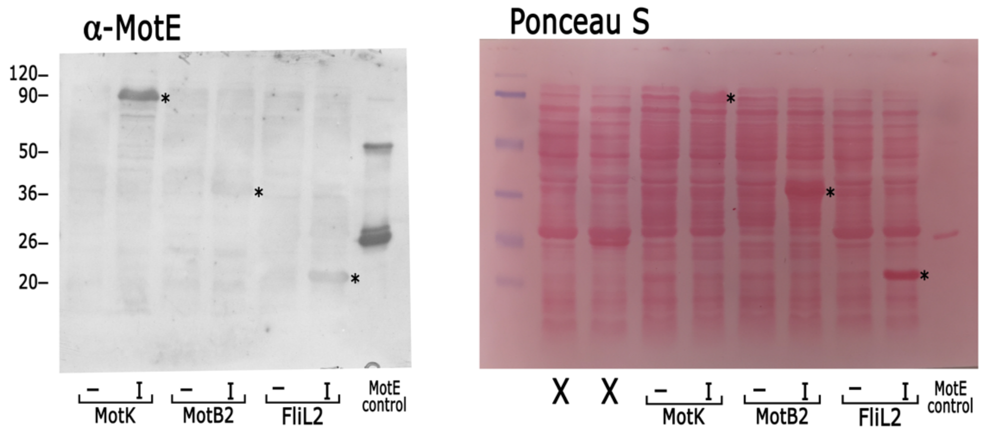

FIG 5 RAW. Image labeled as α-MotE is a scan of an X-ray film. Image labeled as Ponceau S is from a digital picture.

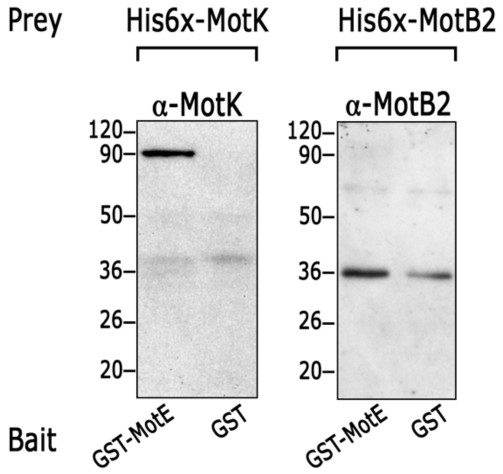

FIG 6

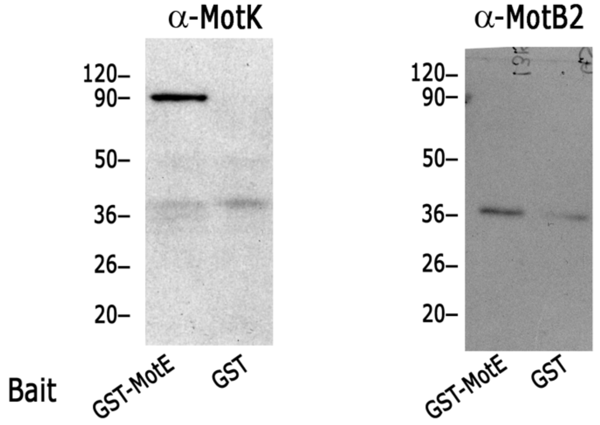

FIG 6 RAW. Images are scans of X-ray films.

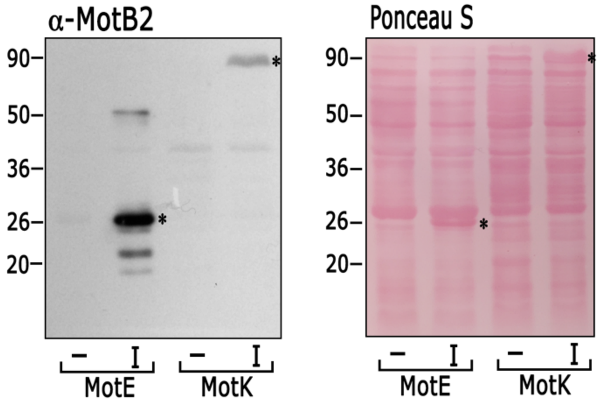

FIG 7

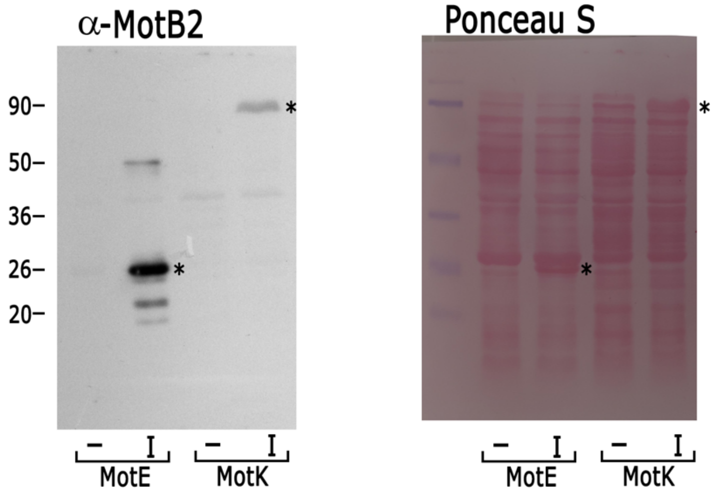

FIG 7 RAW. Image labeled as α-MotB2 is a scan of an X-ray film. Image labeled as Ponceau S is from a digital picture.

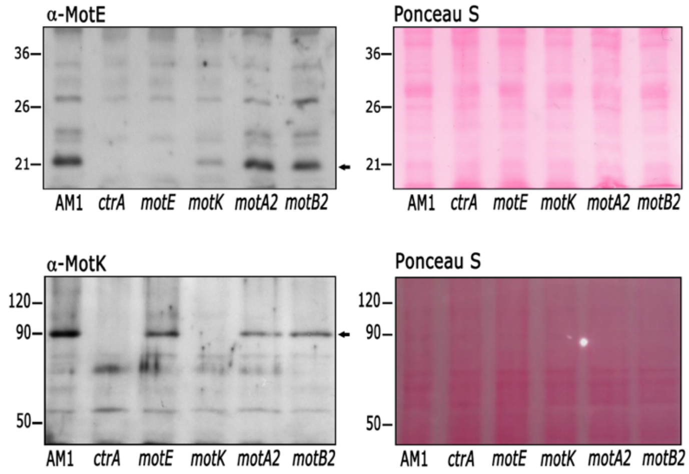

FIG 8

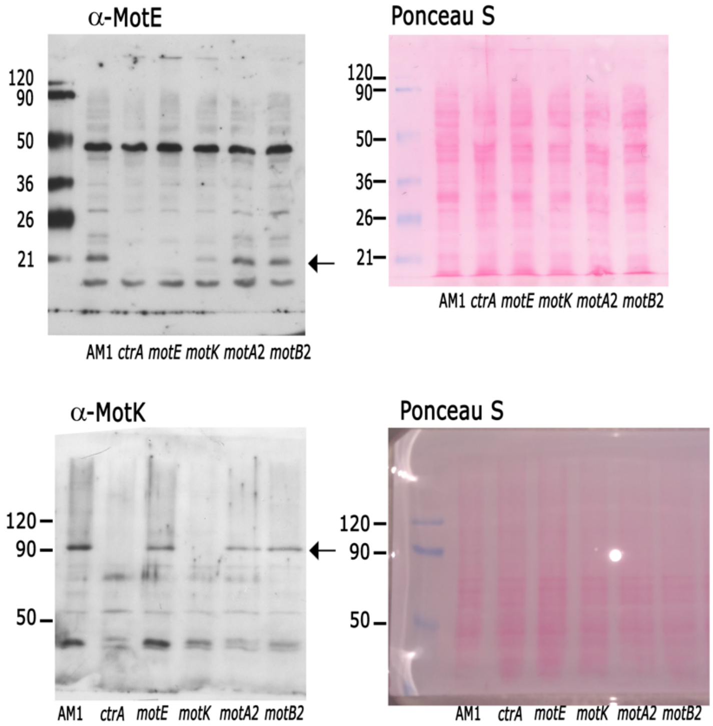

FIG 8 RAW. Images labeled as α-MotE and α-MotK are scans of X-ray films. Images labeled as Ponceau S are pictures from a digital camara. The flash seen on the image below at the right was slightly smoothed for the final figure. As mentioned in the figure legend of Fig 3, non-specific recognition of other polypeptides occurred with both antibodies; however, the identity of MotE and MotK was determined using the proper mutant strain, see Fig 3.

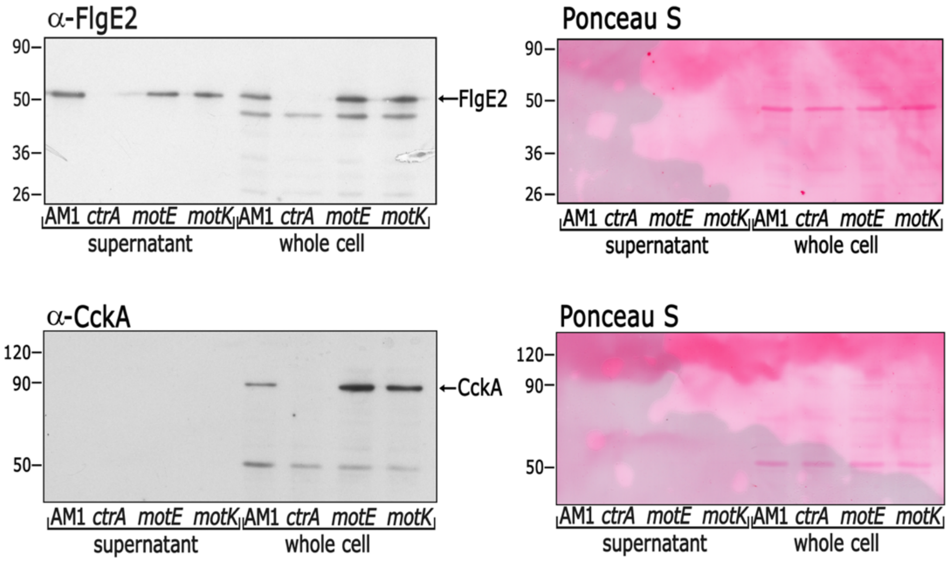

FIG S3

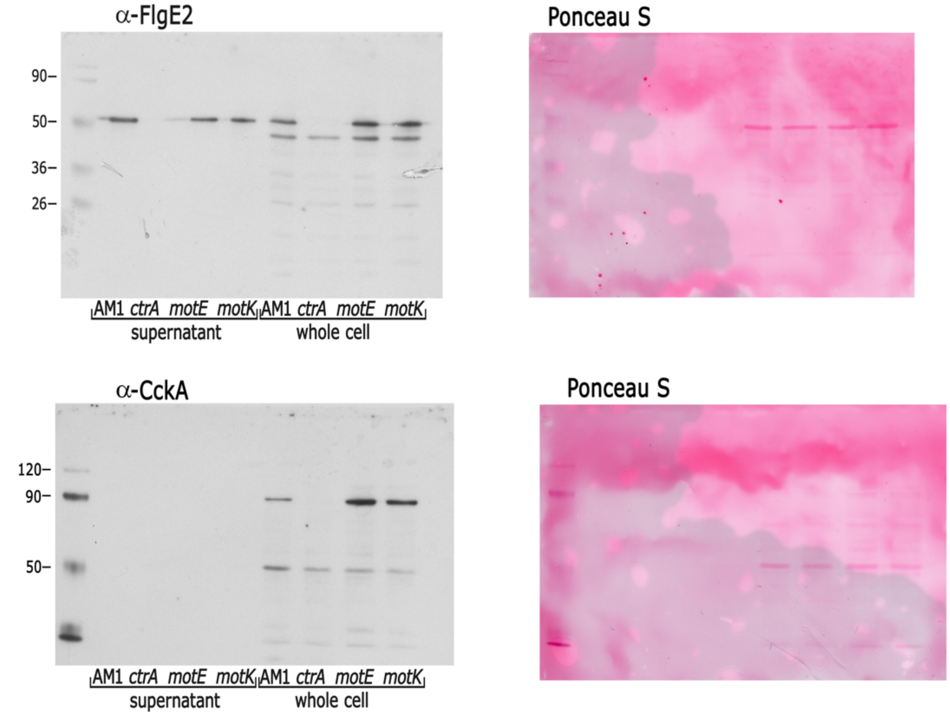

FIG S3 RAW. Images labeled as α-FlgE2 and α-CckA are scans from X-ray films. Images labeled as Ponceau S are pictures from a digital camara.

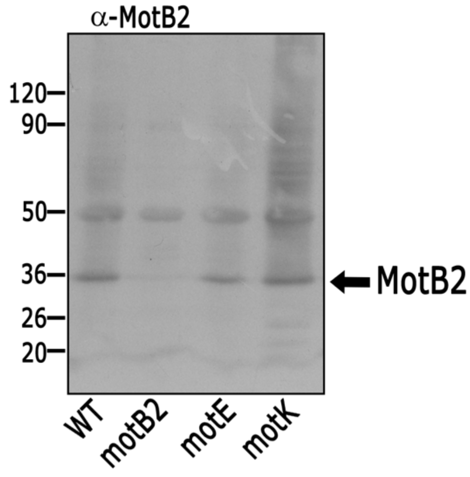

FIG S4

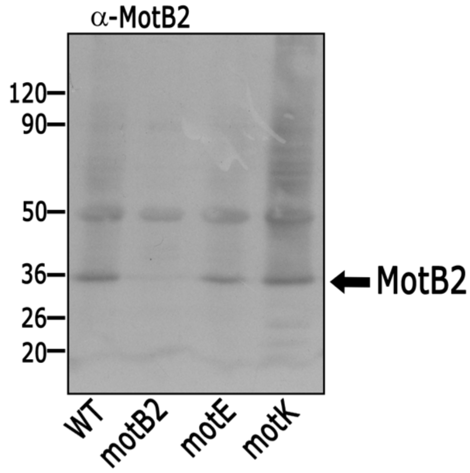

FIG S4 RAW. Scan of an X-ray film.
